# Haem iron reshapes colonic luminal environment: impact on mucosal homeostasis and microbiome through aldehyde formation

**DOI:** 10.1186/s40168-019-0685-7

**Published:** 2019-05-06

**Authors:** Océane C. B. Martin, Maïwenn Olier, Sandrine Ellero-Simatos, Nathalie Naud, Jacques Dupuy, Laurence Huc, Sylviane Taché, Vanessa Graillot, Mathilde Levêque, Valérie Bézirard, Cécile Héliès-Toussaint, Florence Blas Y. Estrada, Valérie Tondereau, Yannick Lippi, Claire Naylies, Lindsey Peyriga, Cécile Canlet, Anne Marie Davila, François Blachier, Laurent Ferrier, Elisa Boutet-Robinet, Françoise Guéraud, Vassilia Théodorou, Fabrice H. F. Pierre

**Affiliations:** 1 INRA, ToxAlim (Research Centre in Food Toxicology), Université de Toulouse, INRA, ENVT, INP-Purpan, UPS, Toulouse, France; 2 INRA, UMR Physiologie de la Nutrition et du Comportement Alimentaire, AgroParisTech, INRA, Université Paris-Saclay, Paris, France; 3 INSA Toulouse LISBP MetaToul, Toulouse, France; 4 ADIV, 10 Rue Jacqueline Auriol, 63039 Clermont-Ferrand, France

**Keywords:** Lipoperoxidation, Barrier function, Metabolites, Dysbiosis, Meat

## Abstract

**Background:**

The World Health Organization classified processed and red meat consumption as “carcinogenic” and “probably carcinogenic”, respectively, to humans. Haem iron from meat plays a role in the promotion of colorectal cancer in rodent models, in association with enhanced luminal lipoperoxidation and subsequent formation of aldehydes. Here, we investigated the short-term effects of this haem-induced lipoperoxidation on mucosal and luminal gut homeostasis including microbiome in F344 male rats fed with a haem-enriched diet (1.5 μmol/g) 14–21 days.

**Results:**

Changes in permeability, inflammation, and genotoxicity observed in the mucosal colonic barrier correlated with luminal haem and lipoperoxidation markers. Trapping of luminal haem-induced aldehydes normalised cellular genotoxicity, permeability, and ROS formation on a colon epithelial cell line. Addition of calcium carbonate (2%) to the haem-enriched diet allowed the luminal haem to be trapped in vivo and counteracted these haem-induced physiological traits. Similar covariations of faecal metabolites and bacterial taxa according to haem-induced lipoperoxidation were identified.

**Conclusions:**

This integrated approach provides an overview of haem-induced modulations of the main actors in the colonic barrier. All alterations were closely linked to haem-induced lipoperoxidation, which is associated with red meat-induced colorectal cancer risk.

**Electronic supplementary material:**

The online version of this article (10.1186/s40168-019-0685-7) contains supplementary material, which is available to authorized users.

## Background

Recently, the World Organization of Health (WHO) classified processed meat intake as “carcinogenic for humans” and red meat intake as “probably carcinogenic” [1]. Several red meat components have been suspected to explain the link with colorectal cancer risk, including animal fat and proteins, heterocyclic amines, *N*-nitroso compounds, and haem iron [2, 3]. Among these compounds, we previously showed that haem iron is the major actor in meat-induced promotion of colorectal cancer without additive or synergistic effects of heterocyclic amines and endogenous *N*-nitroso compounds [4]. Moreover, haem iron intake was recently positively associated with colorectal and colon adenoma risk in a large prospective cohort study [5].

In rodent models, the promotion of colon carcinogenesis by red meat consumption is at least in part explained by haem-induced lipoperoxidation [4, 6–8]. Dietary haem iron catalyses lipid oxidation, leading to the production of alpha-beta unsaturated aldehydes (alkenals), such as 4-hydroxynonenal (HNE) from n-6 fatty acids [9]. This formation of aldehydes correlates with the promotion of preneoplastic lesions in rats and of adenoma in *Min* mice [4]. Furthermore, limiting aldehyde formation by supplementing the diet with calcium carbonate limits the haem-induced promotion of carcinogenesis in rats [6–8]*.* In vitro studies with normal and preneoplastic cells mutated on Apc (adenomatous polyposis coli), an early and frequently mutated gene in colorectal carcinogenesis, indicated that HNE from haem-induced lipoperoxidation selects Apc-mutated cells and enhances the promotion of cancer [10]. Furthermore, haem iron and aldehydes can enhance in vitro cellular inflammatory processes [11], cellular permeability [12], and cellular DNA damage [13, 14]. Interestingly, in vivo, the reduction of gut microbiota by antibiotics prevents haem-induced lipoperoxidation, suggesting a role of the microbiota in the haem-induced formation of aldehydes [15]. Though the effects of haem or aldehydes have already been studied independently on microbiota or colon epithelial cells, to the best of our knowledge, no in vivo study has investigated the short-term effect of dietary haem iron and subsequently formed aldehydes on gut barrier actors as a whole.

As homeostasis of the intestinal mucosal ecosystem results from a subtle interplay between epithelial cells, commensal bacteria, local immune cells, and metabolites, a central challenge in relating food and cancer or food and gut function is to identify both host and microbial interrelated factors that drive the effects of diet. Thus, we used an integrated approach combining microbial community analysis based on microbial 16S rRNA gene sequencing, metabolomics based on ^1^H nuclear magnetic resonance (NMR), and mucosal endpoints in rats to evaluate the impact of haem at a nutritional dose on the colonic luminal environment.

## Methods

### Animal model

Fischer 344 male rats were purchased at 5 weeks from Charles River (Saint-Germain-Nuelles, France). The rats were housed individually in metabolic cages, allowed free access to diet and tap water, in a room kept at a temperature of 22 °C on a 12-h light-dark cycle. After acclimatisation, the rats were randomly allocated into dietary groups; the rats were fed control (CON), calcium carbonate (Ca), haemin (HEM), or haemin+calcium carbonate (HEM-Ca) diets for 14 or 21 days. Body weight was monitored at the beginning, at the middle, and at the end of experimental periods, and food and water intakes were recorded at the end of experiments.

### Experimental diets

All diets were based on a modified low calcium AIN76 diet in a powdered form (UPAE, INRA, France), balanced in iron (haem in haem-enriched diets vs. ferric citrate in control diets), proteins (20% using casein), and lipids (5% using safflower oil). Haem groups (HEM and HEM-Ca) received 1.5 μmol/g diet of haemin (Sigma Chemical), and control groups (CON and Ca) received 0.036% of ferric citrate to balance iron level. Calcium level is critical for the haem promotion of carcinogenesis; calcium was thus excluded from mineral mix but dibasic calcium phosphate was included in all diets at a low concentration of 3.4 g/kg. Calcium groups (Ca and HEM-Ca) received 2% of calcium carbonate (Sigma chemical) in order to chelate haem in the intestinal lumen.

### Faecal and urinary biomarkers

Twenty-four-hour faecal pellets and urine samples were collected under each metabolic cage to assess faecal haem, faecal thiobarbituric reactive substances (TBARs), and urinary 1,4 dihydroxynonene mercapturic acid (DHN-MA) as described previously [7, 16]. Faecal waters were prepared from 0.5 g of fresh faeces homogenised in 1 ml of distilled water and 50 μl of butylated hydroxytoluene (Sigma) 0.45 M, using Fast-Prep® (MP Biomedicals, Illkirch, France) for 3 cycles of 30 s at 6 m s^−1^. After centrifugation at 5,500*g* for 20 min, faecal water (supernatant) was collected and kept at − 20 °C until use.

### In vivo assessment of colon mucosa inflammation, permeability, and genotoxicity

Colonic mucosa inflammation was evaluated as described previously by myeloperoxidase activity (MPO) and confirmed by measuring the IL-1β and IL-10 cytokines using commercial kits according to the manufacturers’ protocols [17].

Colonic paracellular permeability was evaluated using 51-chromium-labelled ethylenediamine tetra-acetic acid (^51^Cr-EDTA; Perkin Elmer Life Science, Paris, France). After 14 days of experimental diets, rats received an administration of ^51^Cr-EDTA (25.9 kBq) diluted in 0.5 ml of saline by oral gavage. Rats were then placed in metabolic cages, and radioactivity in urine was measured with a gamma counter (Cobra II; Packard, Meriden, CT, USA) after 24 h. Permeability to ^51^Cr-EDTA was expressed as the percentage of total radioactivity administered.

#### Mucosal genotoxicity

Colon mucosa genotoxicity was evaluated by alkaline comet assay. Cells were collected by scraping the mucosa and stored in NaCl 0.075 M/EDTA 0.024 M buffer at pH 7.5 before slow freezing at − 80 °C. After counting, cells were embedded in 0.7% Low Melting Point Agarose (Sigma) and laid on CometAssay® HT slides (Trevigen) in triplicate. Slides were immersed overnight in a lysis solution (NaCl 2.5 M/EDTA 0.1 M/Tris 10 mM pH 10/DMSO 10%/Triton 1%). Then, after 40 min for unwinding in electrophoresis buffer (EDTA 1 mM/NaOH 0.3 M, pH 13), the slides were transferred into an electrophoresis tank at 28 V (resulting in 0.8 V/cm on the platform) for 24 min in buffer (EDTA 1 mM/NaOH 0.3 M). Finally, slides were immersed in a PBS solution for neutralisation and cells were fixed using cold absolute ethanol. For DNA staining, 2 μg/ml of ethidium bromide is added on each sample. Fifty cells per slide and 2 slides per sample were analysed using a Nikon 50i fluorescence microscope equipped with a camera and the Komet 6.0 software. The extent of DNA damage was evaluated for each cell through the measurement of intensity of all tail pixels divided by the total intensity of all pixels in head and tail of comet. The median from these 100 values was calculated and named as % tail DNA. The experiment was done in triplicate.

#### Expression of genes involved in aldehyde detoxification, inflammation, and paracellular permeability

Total RNAs were extracted from tissue using the RNeasy plus Mini kit (Qiagen, France) according to the manufacturer’s instructions. RNA samples were reverse transcribed using the iScript cDNA Synthesis kit (Biorad, France). Amplifications were carried out using a ViiA7 Real-Time PCR System (Applied Biosystems, Forster City, CA, USA). The 384-well plates were prepared by an Agilent Bravo Automated Liquid Handling Platform (Agilent Technologies, Santa Clara, CA, USA). Each well contained a final volume of a 5-μl mix: 2.5 μl of iQ SYBR Green Supermix (Biorad, France) used as a fluorescent dye, 1.5 μl of each primer set, and 1 μL of cDNA material. Thermal cycling conditions were as follows: 3 min denaturation at 95 °C followed by 40 cycles at 95 °C for 15 s, 15 s at 60 °C, 15 s at 72 °C, and a melting curve. Data were collected using the Quant-Studio Real time PCR Software v1.1 (Applied Biosystems). Results were normalised with the housekeeping genes TATA-box binding protein (TBP) and 18S and expressed as absolute abundance/gene copy number (delta Ct method). Sequences of primers designed for rat cells are listed in Additional file 1: Table S1a.

### Assessment of aldhehyde detoxication, inflammation, permeability, genotoxicity, and ROS formation after faecal water treatment of murine colon epithelial cells

#### Cellular model

*Apc*^+/+^ (derived from C57BL/6 J mice) colon epithelial cells [4] express the heat-labile SV40 large T antigen (AgT tsa58) under the control of an IFNγ-inducible promoter. The Apc^+/+^ cell line expressed cytokeratin 18, a marker of their epithelial phenotype [18]. The culture conditions affected cell proliferation due to the thermolabile tsA58 T antigen, which confers conditional immortalization: at 33 °C with IFNγ, the large T antigen is active and drives cellular proliferation, and at 37 °C, the temperature-sensitive mutation yields an inactive protein and cells act like non-proliferating epithelial cells. All studies were conducted at the non-permissive temperature (37 °C).

#### Expression of genes involved in aldehyde detoxification, inflammation, and permeability

Normal murine epithelial colonic cells (Apc^+/+^) were treated with filtered faecal waters diluted at 1/160 from rats fed with CON, Ca, HEM, or HEM-Ca diets to assess the expression of genes involved in aldehyde detoxication, cellular inflammation, and paracellular permeability by RT-qPCR. Real-time quantitative PCR (qPCR) were realised as described in the section above with minor changes in the quantitative PCR amplification conditions: a first one-hold stage at 95 °C for 10 min followed by 40 cycles (95 °C for 15 s and 60 °C for 30 s) and a final extending step (95 °C for 15 s, 60 °C for 1 min, and 95 °C for 15 s) for melt curve analysis. Primer sequences designed for mice cells are listed in Additional file 1: Table S1b.

#### Cellular permeability (TEER)

Cells were seeded onto Transwell inserts (Greiner Bio-one®, 3.0 μm pore polyethylene terephthalate membrane insert, 0.6.10^6^ pores/cm^2^) at a density of 260,000 cells/insert in Dulbecco-modified essential medium (DMEM) supplemented with 10% (*v*/*v*) foetal calf serum, 1% (*v*/*v*) penicillin/streptomycin, and 10 U/ml interferon-γ. After seeding, the inserts were transferred into a cellZscope module (NanoAnalytics Münster, Germany) at 5% CO_2_ and at the permissive temperature of 33 °C. After 24 h, the module was transferred to 37 °C (5% CO_2_) without interferon-γ. When TEER was stabilised, the medium was changed for a medium without foetal calf serum. Six hours after, the cells were treated for 24 h with filtered faecal waters diluted at 1/160 from rats fed with CON, Ca, HEM, or HEM-Ca diets. Faecal waters from rats in the CON and HEM groups were additionally incubated or not with polymer resin to trap aldehydes as previously described [4]. Five replicates were performed per condition, and the experiment was performed three times. TEER values were recorded every 40 min, and the results normalised with the value before treatment.

To verify potential differences in cell viability after treatment, cells seeded on inserts were fixed in paraformaldehyde 4% and stained using fluorescent dye Hoechst 33342 (Life Technologies, 0.5 ng/ml in PBS). Apoptotic (fragmented and/or condensed) and alive nuclei were counted using fluorescence microscope (Evos FL Digital Inverted Microscope, AMG) and expressed as percentage of total population (*n* > 500 nuclei).

#### Cellular genotoxicity

Cells were seeded into 24-well plates at 260,000 cells/well in the same medium described above at the permissive temperature of 33 °C. After 24 h, cells were transferred to 37 °C without interferon-γ for 24 h and then treated with native filtered faecal waters diluted at 1/320 from rats fed with CON, Ca, HEM, or HEM-Ca diets and with faecal water from CON and HEM groups additionally incubated with polymer resin to trap aldehydes. After 24 h, the cells were trypsinized and suspended in culture medium with serum and DNA damage was assessed by alkaline comet assay as described in the section above.

#### Reactive oxygen species formation

Intracellular H_2_O_2_ was measured by flow cytometry using H2-DCF-DA (dichlorodihydro-fluorescein diacetate) (Life Technologies). After treatment with filtered native or aldehyde-depleted faecal waters diluted 1/100 for 1 h, the cells were gently trypsinized, stained for 20 min at 37 °C with H2-DCF-DA (10 μM) in HEPES-buffered solution, and next analysed using a MACSQuant Analyzer (Miltenyi Biotec). Menadione (100 μM) (Sigma-Aldrich) was used as a positive control. Each measurement was conducted on 40,000 events in the B1 channel (525 ± 25 nm) and analysed with VenturiOne software.

### ^1^H-NMR metabolomics

#### Faecal extracts preparation

Faecal extracts for NMR spectroscopy and microbiota composition analysis were prepared by homogenising 1 g of frozen faecal pellets three times using a FastPrep® (MP Biomedicals, Illkirch, France) at 6 m s^− 1^ for 30 s. Sixty milligrams of this homogenate were suspended in 1.2 ml of phosphate buffer (0.2 M, pH 7.4) containing 90% D_2_O, 1% (*w*/*v*) of sodium 3-(trimethylsilyl)propionate (TSP), and 0.3 mM NaN_3_. After vortex mixing, samples were centrifuged at 10,000*g* for 10 min at 4 °C. The supernatants were collected and transferred into an NMR tube (outer diameter, 5 mm) pending NMR analysis.

#### Instrumental settings

All ^1^H-NMR spectra from faecal extracts were obtained on a Bruker Ascend 800 Advance III NMR spectrometer (Bruker, France) on the LISBP metabolomics platform (MetaToul) operating at 800.13 MHz for the ^1^H resonance frequency using an inverse detection 5-mm ^1^H-^13^C-^31^P-^15^N cryoprobe (CQPCI) attached to a cryoplatform (preamplifier cooling unit). The ^1^H-NMR spectra were acquired at 298K using the Carr-Purcell-Meiboom-Gill (CPMG) spin-echo pulse sequence with pre-saturation and a total spin-echo delay (2nτ) of 100 ms. A total of 32 transients were collected into 64,000 data points using a spectral width of 15 ppm, a relaxation delay of 5 s, and an acquisition time of 2.72 s.

#### Data analysis

Data were analysed by applying an exponential window function with a 0.3-Hz line broadening prior to Fourier transformation. The resultant spectra were phased, baseline corrected, and calibrated to TSP (δ 0.00) manually using Mnova NMR (v9.0, Mestrelab Research). The spectra were subsequently imported into MatLab (R2014a, MathsWorks, Inc.). The region containing the water resonance (*δ* 4.6–5.2 ppm) was removed, and the spectra were normalised to the probabilistic quotient [19] and aligned using a previously published function [20]. All data were analysed using full-resolution spectra. Data were mean-centred and scaled using the unit variance scaling prior to analysis using projection on latent structure-discriminant analysis (O-PLS-DA). A first PLS-DA model was build using the mixOmics R package (6.1.1 version) [21–23] using all four treatment groups. Pairwise O-PLS-DA models were then constructed to compare the groups 2 by 2. ^1^H-NMR data were used as independent variables (*X* matrix) and regressed against a dummy matrix (*Y* matrix) indicating the class of samples (CON vs HEM, CON vs Ca, HEM vs HEM-Ca) [24]. PLS-derived models were evaluated for goodness of prediction (Q2Y value) using eightfold cross-validation. Parameters of the final models are indicated in the figure legends. To identify metabolites responsible for discrimination between the groups, the O-PLS-DA correlation coefficients (r2) were calculated for each variable and back-scaled into a spectral domain, so that the shape of NMR spectra and the sign of the coefficients were preserved [25]. The weights of the variables were colour-coded, according to the square of the O-PLS-DA correlation coefficients. Correlation coefficients extracted from significant models were filtered so that only significant correlations above the threshold defined by Pearson’s critical correlation coefficient (*P* < 0.05; |r2| > 0.49) were considered significant. For illustration purposes, the area under the curve of several signals of interest was integrated and statistical significance was tested using *t* test.

### SCFA assay in faeces

All the organic acids were extracted by vigorous homogenisation with ultrapure water followed by centrifugation (14,000*g*, 15 min at 4 °C). The SCFA in the supernatants was then derivatised by esterification and analysed with a gas chromatograph equipped with a capillary column (30 m, 0.32 mm ID; RestekRtx 502.2) and fitted with a flame ionisation detector using a modification of the method of Kristensen et al. [26]. The amounts of SCFA were determined by external standards with reference to internal standards.

### Microbial community analysis

Genomic DNA was obtained from faecal extracts using the ZR Faecal DNA Miniprep^TM^ kit (Zymo Research), and DNA quantity was determined using a TECAN Fluorometer (Qubit® dsDNA HS Assay Kit, Molecular Probes).

#### 16S rRNA gene amplification and amplicon sequencing

The V3-V4 hypervariable region of the 16S rRNA gene was amplified by PCR. The forward PCR primer **5′CTT TCC CTA CAC GAC GCT CTT CCG ATC T**AC GGR AGG CAG CAG3′ was a 43-nuclotide fusion primer consisting of the 28-nt illumina adapter (designed by bold font) and the 14-nt broad range bacterial primer 343F. The reverse PCR primer 5**′GGA GTT CAG ACG TGT GCT CTT CCG ATC T**TA CCA GGG TAT CTA ATC CT3′ was a 47-nuclotide fusion primer consisting of the 28-nt illumina adapter (designed by bold font) and the 19-nt broad range bacterial primer 784R.

The PCR mix contained MTP Taq DNA polymerase (SIGMA, 0,05 U/μl), 200 μM of each DNTP (SIGMA, premix), and 0,5 μM of each primer. After initial denaturation at 94 °C for 60 s in CFX-96 Thermal Cycler (Bio-Rad), 30 cycles were run with 60 s denaturation at 94 °C, 60 s annealing at 65 °C, and 60 s at 72 °C, round ended with 10 min extension at 72 °C. Amplification quality (length, quantity, and specificity) was verified using the Agilent 2200 TapeStation System (High Sensitivity D1000 ScreenTape assay) and AATI Fragment Analyser at the GeT (Genomic and Transcriptomic, TRIX, and PlaGe) platforms in Toulouse. Because MiSeq enables paired 250-bp reads, the ends of each read are overlapped and can be stitched together to generate extremely high-quality, full-length reads of the entire V3 and V4 region in a single run. Single multiplexing was performed using home-made 6 bp index, which were added to R784 during a second PCR with 12 cycles using forward primer (AAT GATACGGCGACCACCGAGATCTACACTCTTTCCCTACACGAC) and reverse primer (CAAGCAGAAGACGGCATACGAGAT-index-GTGACTGGAGTTCAGACGTGT). The resulting PCR products were purified and loaded onto the Illumina MiSeq cartridge according to the manufacturer instructions. The quality of the run was checked internally using PhiX, and then, each pair-end sequences were assigned to its sample with the help of the previously integrated index. Each pair-end sequences were assembled using Flash software [27] using at least a 10-bp overlap between the forward and reverse sequences, allowing 10% of mismatch. The lack of contamination was checked with a negative control during the PCR (water as template). The quality of the stitching procedure was controlled using four bacterial samples that are run routinely in the sequencing facility in parallel to the current samples.

#### 16S rRNA gene analysis

High quality filtered reads (2,502,588 reads) were further processed using FROGS pipeline (Find Rapidly OTU with Galaxy Solution) to obtain OTUs and their respective taxonomic assignment thanks to Galaxy instance (https://galaxy-workbench.toulouse.inra.fr) [28]. Initial FROGS pre-process step allowed to select overlapped reads with expected length without *N*, yielding to 1,886,283 pass-filter reads (an average of 60,000 reads per sample). Swarm clustering method was applied by using a first run for denoising with a distance of 1 and then a second run for clustering with an aggregation maximal distance of 3 on the seeds of first swarm [29], yielding to 267,558 clusters (an average of 10,000 per sample). Putative chimaeras were removed using VSEARCH combined to cross-validation (GitHub repository. Doi 10.5281/zenedo.15524), yielding to 189,803 clusters (an average of 6400 per sample). Cluster abundances were filtered at 0.005% [30] and/or had to be present at least in three samples, yielding to 332 final clusters (an average of 212 clusters per sample) corresponding to 1,425,084 final valid reads (an average of 44,534 valid reads per sample). One hundred percent of clusters were affiliated to OTU by using a silva123 16S reference database and a taxonomic multi-affiliation procedure (Blast+with equal multi-hits [31]). Since rarefaction has shown to result in high rates of false-positive tests for differential abundance, counts were not rarefied [32]. Richness and diversity indexes of bacterial community, as well as clustering and ordinations, were computed using the Phyloseq package (v 1.19.1) in RStudio software [23, 33]. Within sample community alpha diversity was assessed by observed diversity (i.e. sum of unique OTUs per sample) and Simpson index, abundance-based richness indices. Divergence in community composition between samples was quantitatively assessed by calculating weighted UniFrac (abundance and phylogenetic relation) distance matrices. Unconstrained ordination was visualised using multidimensional scaling (MDS) and hierarchical clustering (complete linkage combined with wUniFrac distance) and compared using Adonis test (9999 permutations).

In order to evaluate differential abundance in response to diet and identify important taxa modulated by haem and associated to lipoperoxidation status, OTUs were agglomerated at the species rank, reducing the taxon list to 122. Differentially abundant taxa were identified by characterising the difference between two different diets (multivariate analysis, Kruskall-Wallis non-parametric pairwise comparisons) using LEfSe algorithm with an alpha value of 0.01 and a threshold on the logarithmic LDA score for discriminative features of 3 [34] Univariate differential abundance of taxa was also tested using a negative binomial noise model for over dispersion as implemented in the R package DESeq2 (v1.14.1, [32, 35]). In order to identify taxa altered by haem, LRT method was first applied to select significantly affected taxa across the 4 diets (86 taxa) before applying a pairwise post-test comparison (contrast/Wald test) to sort taxa altered by haem as compared to control diet (39 taxa). In parallel, a 2 × 2 factor design combined with a Wald test was applied in order to identify taxa for which haem effect changed across calcium exposition (interaction term). On the 33 final taxa corresponding to the interaction term, 12 taxa were selected because the addition of calcium in haem-enriched diet restored their initial observed level in the control diet. Taxa were considered significantly differentially abundant between diets if their adjusted *P* value was below 0.01 and if estimated change was log2FC > |1.5|. Tests were corrected for multiple inferences using the Benjamini-Hochberg method to control the false discovery rate. The sequences used for analysis can be found in the MG-RAST database under the project name “Haem_calcium”, with the following accession numbers: mgp89255.

### Integrative analysis of two or three datasets using rCCA or extended sGCCA respectively

Regularised canonical correlations analysis (rCCA) [36] or extended sparse generalised canonical analysis (sGCCA named DIABLO) [37] was performed using the R package mixOmics (v 6.1.1) in order to improve the representation of the links between bacterial taxa, metabolite signatures, physiological traits (metadata), and most importantly, lipoperoxidation status. Both of these supervised multiblock approaches (two or three datasets), for which generic recommended frameworks were applied [36, 37], are able to maximise common or correlated information into a single exploratory analysis. Prior to integrative analysis with other datasets, the microbiota dataset was processed according to the multivariate statistical mixMC framework, which included data processing using Total Sum Scaling (TSS) normalisation and log-ratio transformation [38].

### Statistical analysis

Results are expressed as mean ± SEM unless otherwise stated. For in vivo experiments, *N* refers to the number of animals per group used for in each experiment. The significance of differences between experimental groups was determined by ANOVA with Holm-Sidak multiple comparison post-test, or the Kruskal-Wallis non-parametric ANOVA with Dunn’s multiple comparison post-test as appropriate (Prism 6, Graph Pad Software). Two-side analyses were used throughout, and *P* < 0.05 was considered significant.

## Results

### Haem iron-induced lipoperoxidation is associated with increased markers of colonic inflammation, genotoxicity, and permeability in vivo

**Fig. 1:**
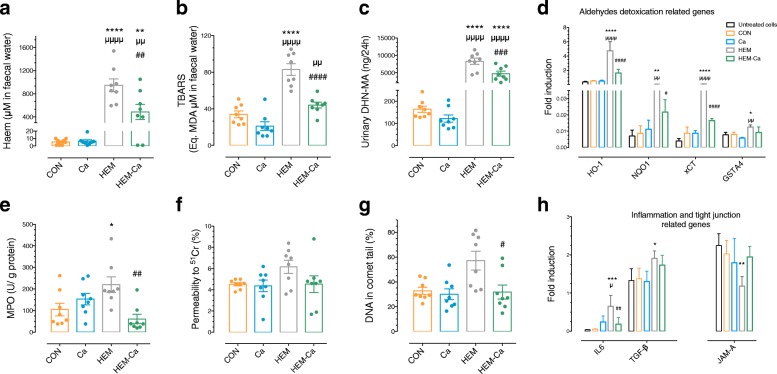
In vivo and ex vivo effects of the addition of haem and/or calcium carbonate on faecal, mucosal, and cellular biomarkers. After 21 days of experimental diets, effect on (**a**) faecal haem, (**b**) faecal TBARs, (**c**) urinary DHN-MA, (**d**) ex vivo expression of aldehyde detoxication-related genes, (**e**) colonic myeloperoxidase activity, (**f**) permeability, (**g**) DNA damage, and (**h**) ex vivo expression of cytokines and JAM-A genes. CON, control diet; Ca, control diet + calcium carbonate; HEM, haem-enriched diet; HEM-Ca, haem-enriched diet + calcium carbonate. Values are presented as means ± SEM; *n* = 8 for in vivo and *n* = 3 to 4 for ex vivo. ANOVA, Holm-Sidak’s multiple comparison test: *versus CON, ^μ^versus Ca, ^#^versus HEM

The addition of dietary haem for 21 days significantly increased luminal faecal haem compared to rats fed with the CON and Ca diets (*P* < 0.0001 and *P* = 0.001, respectively, Fig. 1a). Accordingly, the rats in the HEM group had significantly more lipoperoxidation products in faecal water measured by faecal TBARs and urinary DHN-MA than the rats in the CON group (all *P* < 0.0001, Fig. 1b, c). By exposing cultured colonic epithelial cells to the haem and lipoperoxidation product-enriched rat faecal waters*,* we also observed a significant increase in the expression of aldehyde detoxication-related genes (Fig. 1d). These higher luminal lipoperoxidation levels were associated with higher mucosal inflammation markers as revealed by the increased colonic myeloperoxidase activity levels observed in vivo (*P* = 0.03, Fig. 1e) and increased gene expression of cytokines IL-6 and TGF-β ex vivo (*P* < 0.0008 and *P* = 0.048, respectively, Fig. 1h). In addition, haem induced greater ^51^Cr-EDTA permeability in vivo (Fig. 1f), which correlated with the significant underexpression of the junctional adhesion molecule-A (JAM-A) gene ex vivo (*P* = 0.0082, Fig. 1h). Interestingly, rats fed with the HEM diet exhibited higher DNA damage in the colonic mucosa than the rats fed with the CON diet (Fig. 1g). Moreover, a second in vivo study allowed us to confirm faecal, urinary, and colonic changes in response to HEM diet and to observe their occurrence as early as 14 days after starting the HEM diet (Additional file 2: Figure S1).

### In vivo restriction of luminal haem iron bioavailability by the addition of dietary calcium carbonate alleviates lipoperoxidation and associated barrier defects

The addition of calcium carbonate to the HEM diet (HEM-Ca diet) normalised in vivo luminal haem levels (*P* = 0.018 vs. HEM diet, Fig. 1a), haem-associated lipoperoxidation biomarkers (all *P* < 0.001, Fig. 1b, c), and mucosal inflammation, permeability, and DNA damage (*P* = 0.008, *P* = 0.23, and *P* = 0.04, respectively, Fig. 1e–g).

### Ex vivo trapping of haem-induced luminal aldehydes alleviates epithelial barrier defects

**Fig. 2:**
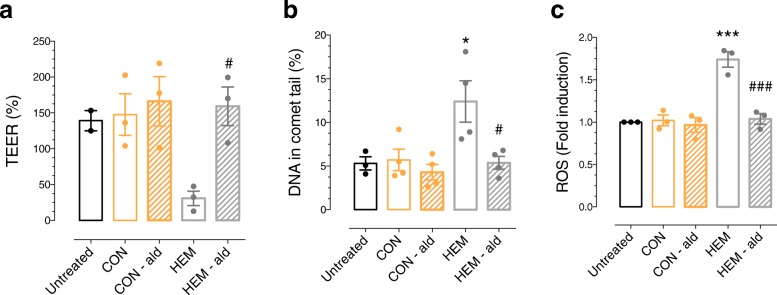
Ex vivo effect of haem-enriched diet. Effect of faecal water from rats fed with control (CON) and haem-enriched (HEM) diets, before or after treatment with a polymer resin carrying hydrazine functional groups, which trap aldehydes, on (**a**) cellular trans-epithelial resistance, (**b**) DNA damage, and (**c**) reactive oxygen species formation on murine colonic epithelial cells. CON, control diet; HEM, haem-enriched diet; -ald, after aldehyde trapping. Values are presented as means ± SEM; *n* = 3 to 4*. P* < 0.05 using one-way ANOVA. Holm-Sidak’s multiple comparison test: *versus CON, ^#^versus HEM

The capability of aldehydes resulting from in vivo haem-induced lipoperoxidation to alter cellular permeability, genotoxicity, and ROS formation was assessed in vitro on colonic epithelial cells. The efficiency of aldehyde trapping in faecal water by the polymer resin carrying hydrazine functional groups had been validated in previous studies [4, 39]. In the present study, aldehyde trapping significantly decreased TBARs in faecal water by 60% in the CON group (37 ± 10 vs. 15 ± 7 eq. MDA before and after treatment, respectively) and 68% in the HEM group (81 ± 18 vs. 26 ± 7 eq. MDA before and after treatment, respectively). Colonic cells treated with faecal water from rats fed with the HEM diet exhibited significantly lower trans-epithelial resistance, reflecting increased cellular permeability (Fig. 2a) and higher levels of DNA damage (Fig. 2b) and reactive oxygen species formation (Fig. 2c) compared to cells treated with faecal water from rats fed with the control diet. Under these dilution conditions, faecal water treatment did not induce any difference in viability nor in the percentage of apoptotic nuclei (CON group: 12.9 ± 3.1% vs. 10.4 ± 2.1% before and after aldehyde treatment; HEM group: 11.8 ± 3.3% vs. 13.7 ± 3.2% before and after aldehyde treatment; *P* = 0.181). When the faecal waters from rats fed with the haem-enriched diet were treated with the polymer resin for aldehyde removal, the parameters were normalised, strongly suggesting aldehyde involvement (Fig. 2).

### Haem iron intake is associated with modified faecal metabolic profiles and calcium carbonate supplementation partially normalises haem iron-induced metabolic perturbations

**Fig. 3:**
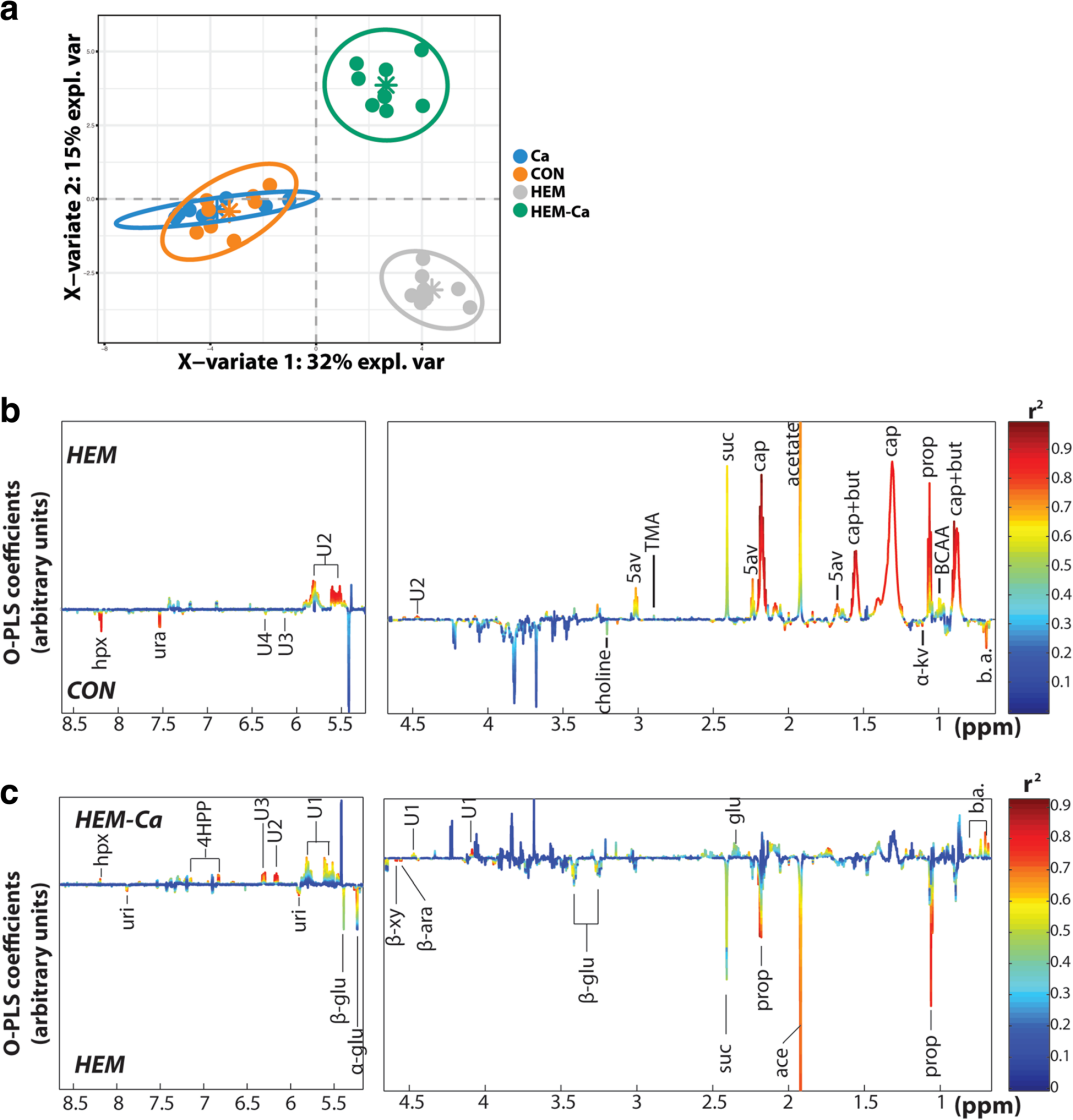
Metabolic profiling in faecal water of rats. **a** PLS-DA score plot. **b**, **c** Plot of O-PLS-DA coefficients related to the discrimination between ^1^H-NMR spectra for rats treated with **b** HEM (top) vs. Con (bottom), and **c** HEM-Ca (top) vs. HEM (bottom). Metabolites are colour-coded according to their correlation coefficient, with red indicating a very strong positive correlation (r2 > 0.7). The direction of the metabolite indicates the group with which it is positively associated as labelled on the diagrams. α-glu, α-glucose; ace, acetate; β-ara, β-arabinose; β-glu, β-glucose; β-xy, β-xylose; b.a., bile acids; BCAA, branched-chain amino acids; but, butyrate; cap, caprylate; hpx, hypoxanthine; prop, propionate; suc, succinate; TMA, trimethylamine; ura, uracil; uri, uridine; U1-4, unknown compounds; 4-HPP, 3-(4-hydroxyphenyl)propionic acid; 5av, 5-aminovalerate

The effect of diet on the faecal metabolic profiles was investigated using NMR-based metabolomics. A typical NMR spectrum for each group is provided in Additional file 2: Figure S2. A global PLS-DA analysis (Fig. 3a) showed clear separation between the metabolic profiles of rats fed with the haem-enriched diets (HEM and HEM-Ca) and those fed with the CON or Ca diets along the first component, demonstrating a strong effect of HEM supplementation on gut microbiota-associated metabolic activity, independent of calcium carbonate supplementation. The second component separated the rats fed with HEM-Ca from the rats fed with HEM, whereas rats fed with the CON and Ca diets were merged.

Pairwise comparison of faecal metabolites in the HEM vs. CON groups confirmed our previous observation that supplementation with dietary haem iron affected both the faecal metabolomic profile and the number of metabolites more markedly than supplementation with calcium carbonate (Q^2^Y = 0.91, R^2^Y = 0.96, *P* = 0.001, Fig. 3b). HEM-fed rats presented lower faecal levels of all bile acids detected (Additional file 1: Table S2a), choline, uracil, and hypoxanthine and higher levels of the short chain fatty acids acetate, propionate, and butyrate; the medium chain fatty acid caprylate (C8:0); the branched chain amino acids leucine, isoleucine, and valine; and of trimethylamine, succinate, and 5-aminovalerate (Additional file 1: Table S2a), which is known as a microbial intermediate product of amino acid degradation.

Finally, the pairwise comparison between the faecal metabolic profiles of the HEM and HEM-Ca groups was also highly significant (Q^2^Y = 0.82, R^2^Y = 0.96, *P* = 0.001, Fig. 3c), with increased levels of five bile acids, glutamate, hypoxanthine, 4-hydroxypropionic acid, and several unknown compounds observed in the faecal extracts of HEM-Ca-fed rats. The rats fed with HEM-Ca also had decreased levels of butyrate, propionate, acetate, succinate, and several carbohydrates, such as α- and β-glucose, β-arabinose, and β-xylose.

**Fig. 4:**
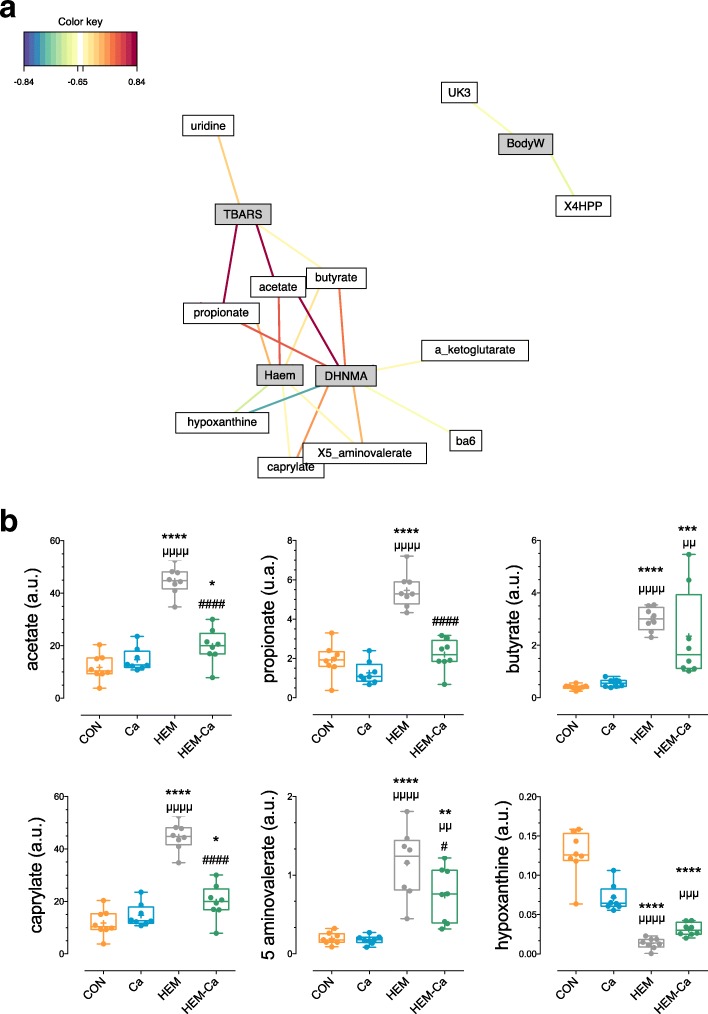
Correlation between metabolomic and physiological data. **a** Relevance networks representing the stronger correlations (higher than 0.65) between 25 metabolites and 12 animal physiological traits (metadata) selected with rCCA. The edge colours indicate the nature of the correlation (positive in red, negative in blue). Metabolites and metadata are represented respectively as white and grey rectangles. **b** Boxplot of the area under the curve of the NMR spectra for selected metabolites that covariated with lipoperoxidation status. *P* < 0.05 using ANOVA. Holm-Sidak’s multiple comparison test: *versus CON, ^μ^versus Ca, ^#^versus HEM. Haem, haem in faecal water; BodyW, body weight; UK3, unknown compound; 4HPP, 3-(4-hydroxyphenyl)propionic acid; ba6, unknown bile acid

Metabolomic data were statistically merged with previously described physiological traits (metadata including permeability, peroxidation, and genotoxicity) using rCCA to unravel specific correlations between the two datasets (Fig. 4a; Additional file 2: Figure S3). We observed that faecal haem and lipoperoxidation product (DHN-MA and TBARs) levels positively correlated mainly with the short-chain fatty acids (SCFAs: acetate, propionate, and butyrate), caprylate, and 5-aminovalerate and negatively correlated with hypoxanthine.

The addition of calcium carbonate to the HEM diet significantly counteracted the haem-induced levels of these metabolites (Fig. 4b). These perturbations in faecal SCFAs were confirmed using a dedicated targeted assay through gas chromatography (Additional file 1: Table S2b).

### Bioavailability of dietary haem iron alters the community structure of the faecal microbiota

**Fig. 5:**
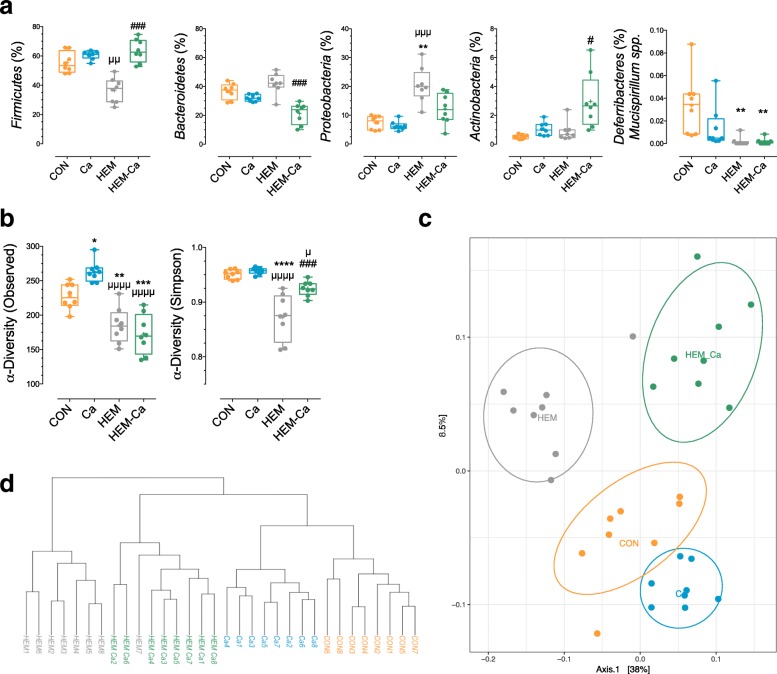
Effect of dietary haem and/or calcium carbonate on the community distribution and diversity of the faecal microbiota as determined by 16S rRNA gene Illumina Miseq sequencing. **a** Relative abundance (%) per phylum according to diet. **b** Richness (α-diversity) measured by observed OTU number and Simpson Index according to diet. **c** Weighted UniFrac Multidimensional Scaling (MDS) plot representing structural changes between diets (β-diversity). **d** Hierarchical clustering based on the wUniFrac distances with complete linkage. CON, control diet; Ca, control diet + calcium carbonate; HEM, haem-enriched diet; HEM-Ca, haem-enriched diet + calcium carbonate. Values are presented as mean ± SEM; *n* = 8. *P* < 0.05 using ANOVA. Holm-Sidak’s multiple comparison test (b) or Kruskal-Wallis test with Dunn’s multiple comparison post-test (c): *versus CON, ^μ^versus Ca, ^#^versus HEM

Faecal microbiota analysis by Illumina sequencing of the 16S rRNA gene revealed profound shifts in the composition of the microbiota according to diet, as evidenced quantitatively at the OTU level by the abundance of the detected phyla (Fig. 5a). Exposure to the HEM diet resulted in a lower abundance of *Firmicutes* (*P* = 0.08 vs. CON) and *Deferribacteres* (*P* = 0.001 vs. CON), as well as promoted *Bacteroidetes* and *Proteobacteria* (*P* = 0.003 vs. CON). Interestingly, these changes, except for the loss of *Deferribacteres* (*P* > 0.99 vs. HEM), were partially reversed by the addition of calcium carbonate to the HEM diet (*P* = 0.003, *P* < 0.0001, and *P* = 0.3 vs. HEM for *Firmicutes*, *Bacteroidetes*, and *Proteobacteria*, respectively).

Though the Ca diet did not obviously alter the quantitative composition of the microbiota at the phylum level compared to the CON diet (Fig. 5a), it significantly promoted the number of observed OTUs, as indicated by the alpha diversity index of richness (*P* = 0.025, Fig. 5b). The number of observed OTUs was significantly decreased in the HEM and HEM-Ca groups (~ 21% loss vs. CON, *P* < 0.01, Fig. 5b), whereas the alpha diversity index of Simpson was clearly decreased only in the HEM group, suggesting that only evenness was partially restored by the addition of calcium carbonate in the haem-enriched diet (*P* = 0.0005 vs. HEM, Fig. 5b). Multidimensional scaling and cluster analysis of weighted UniFrac distances (Fig. 5c, d) revealed that the faecal microbiota of rats clustered separately according to diet, with the HEM diet being the most distant from the other three balanced diets. The corresponding beta diversity obtained using the Adonis test confirmed that diet composition explained roughly 65% of the total variation between samples (*P* < 0.0001, Fig. 5c).

### Taxonomic alterations in faecal microbiota exposed to haem-enriched diet

**Fig. 6:**
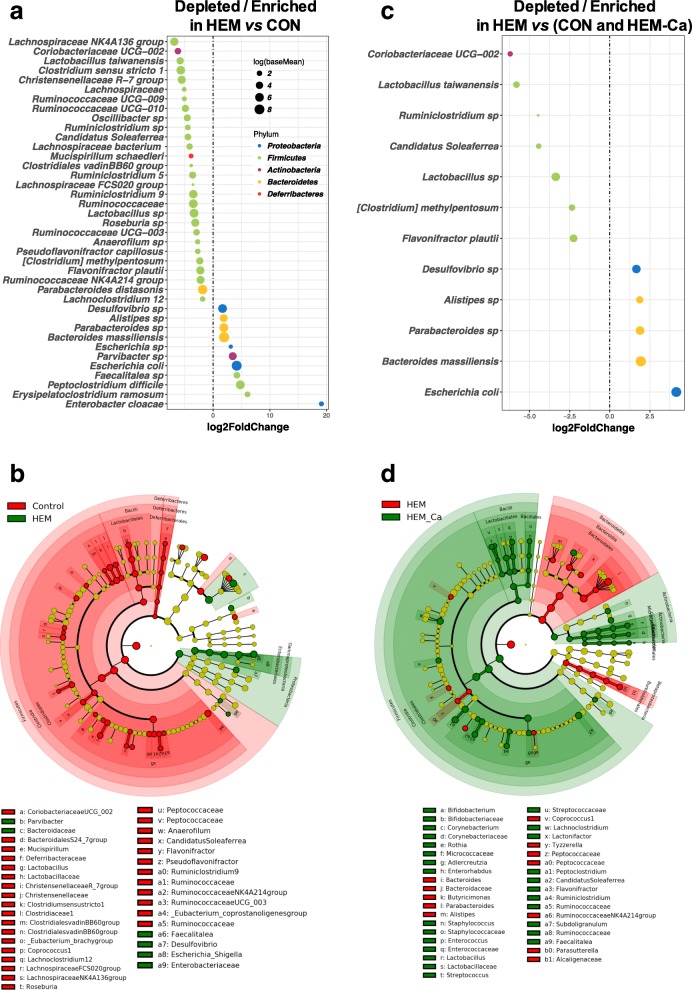
Differentially abundant faecal bacterial taxa in response to the addition of dietary haem and/or calcium carbonate. **a** Classified differentially abundant taxa between rats fed with a haem-enriched diet and control diet. Log2FoldChange (HEM vs. CON) = log_2_(HEM/CON) is plotted on the *X*-axis. Phylum is indicated using colour codes. Features were considered significant if their adjusted post-test *P* value was < 0.01 (haem effect against other three groups). **b** Circular cladogram generated from LEfSe analysis showing the most differentially abundant taxa enriched in microbiota from rats fed with control (red) or haem (green) diets. **c** Classified differentially abundant taxa between rats fed a haem-enriched diet and other diets. The taxa were identified using a 2 × 2 factor design by sorting the interaction term corresponding to haem-affected taxa, for which the addition of calcium restored the initial levels observed in the control diet. Log2FoldChange (HEM vs. (CON and HEM-Ca) = log_2_(HEM/(CON and HEM-Ca)) is plotted on the *X*-axis. Phylum is indicated by colour codes. Features were considered significant if the adjusted *P* value of the interaction term was < 0.01. **d** Cladogram showing the most differentially abundant taxa enriched in microbiota from rats fed with haem (red) or haem-calcium (green) diets. LDA scores > 3 and significance of alpha < 0.01 determined by Kruskal-Wallis test. Corresponding LDA scores are presented in Additional file 2: Figure S4

Over the 122 assigned taxa at the species rank, 86 were significantly affected by the diet (padj < 0.01), among which 39 were related to the impact of the HEM diet (padj < 0.01; Fig. 6a). Supplying a diet containing haem iron was also associated with a relative decrease in the abundance of 28 out of 39 taxa that mainly belong to the *Firmicutes* phylum and more specifically to the *Clostridiales* order (Fig. 6a, b; Additional file 1: Table S3a). Interestingly, the only three *Firmicutes* members that were significantly enriched in the HEM group (i.e. two genera belonging to *Erysipelotrichaceae* and *Peptoclostridium difficile*) were pathobionts [40, 41] (Additional file 2: Figure S5). Three taxa from different families of *Bacteroidetes* (*Bacteroidaceae*, *Pophyromonadaceae*, and *Rikenellaceae*) and four taxa among *Proteobacteria* (three *Enterobacteriaceae* and an unknown species belonging to *Desulfovibrio* genus) were overabundant (Fig. 6a). Numerous major changes observed in response to exposition to dietary haem were similar between the exposition of the CON (Fig. 6b) and the combined expositions of haem and calcium (Fig. 6c, d). Among these major observed changes, *Defferibacteres* represented by *Mucispirillum schaedleri* was the only phylum affected by haem regardless of whether calcium carbonate was added (Fig. 5a).

### Identification of taxa that covariated with lipoperoxidation status

**Fig. 7:**
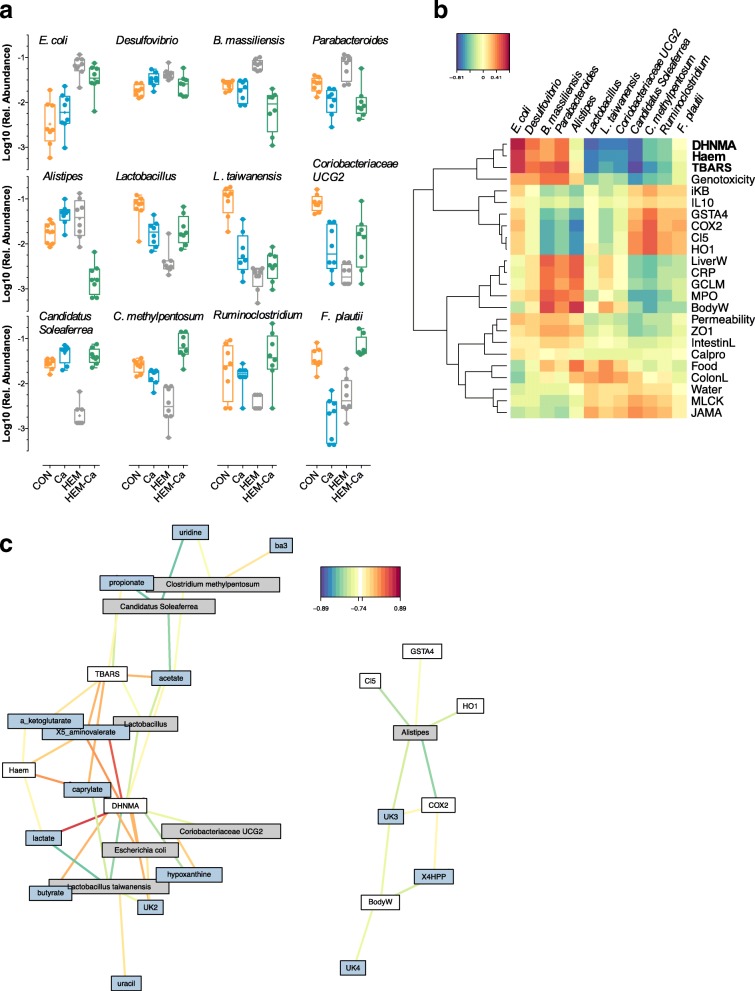
Bacterial communities that covariated with lipoperoxidation status and metabolites. **a** Relative abundance of bacterial taxa (12) that strongly covariated with haem lipoperoxidation status. **b** CIM of 12 previously selected bacterial taxa and 19 physiological traits (metadata) selected with rCCA. **c** Relevance network of 12 bacterial taxa, metadata, and metabolites selected with DIABLO framework. Only associations with an absolute correlation > 0.75 are represented. Red and blue colours indicate regions where bacterial taxa, metadata, and metabolites are highly positively and negatively correlated, respectively. CON, control diet; Ca, control diet + calcium carbonate; HEM, haem-enriched diet; HEM-Ca, haem-enriched diet + calcium carbonate; DHNMA, urinary 1,4 dihydroxynonenal; Haem, haem in faecal water; TBARS, faecal thiobarbituric reactive substances; Genotoxicity, DNA damage by comet assay; iKB, ikappaB; IL10, IL10 in serum; GSTA4, gene expression of glutathione S-transferase alpha 4; COX2, cyclooxygenase2; Cl5, claudin 5; HO1, Haemoxygenase1; LiverW, weight of liver; CRP, C-reactive protein; GCLM, glutamate-cysteine ligase modifier; MPO, myeloperoxidase; BodyW, body weight; Permeability, Cr51 permeability; ZO1, tight junction protein 1; IntestinL, intestine length; Calpro, Calprotectin; Food, food intake; Colon L, colon length; Water, water intake; MLCK, myosin light-chain kinase; JAMA, junctional adhesion molecule; UK2-4, unknown compounds; 4HPP, 3-(4-hydroxyphenyl)propionic acid; ba3, unknown bile acid

By performing a two-factor analysis, we sorted 12 taxa significantly altered by haem and calcium (the interaction term, padj < 0.01), for which haem-induced alterations were restored by the HEM-Ca diet (Figs. 6c and 7a; Additional file 1: Table S3b). Using supervised approaches allowing to integrate multi-datasets, correlations between these 12 taxa, the previously described physiological traits and metabolites were maximised in order to reconstruct taxa-associated network (Fig. 7b, c; Additional file 2: Figure S6): Haem iron bioavailability in faecal water (“Haem”) was significantly associated with the relative increased abundance of two members of *Proteobacteria* (*Escherichia coli* and *Desulfovibrio spp*.) and two taxa belonging to *Bacteroidales* order (unclassified species of *Parabacteroides* and *Bacteroides massiliensis*) (Fig. 7b). In contrast, relative abundances of *Lactobacillus* at the genus level (two taxa), of a member of *Coriobacteriaceae* (UCG-002), and of three taxa belonging to *Ruminococcaceae* (*Candidatus Soleaferrea*, *Clostridium methylpentosum*, and unclassified species of *Ruminoclostridium*) were negatively associated with haem iron in faecal waters (Fig. 7b). As expected, stronger correlations between microbial taxa and physiological metadata obtained through rCCA refer to lipoperoxidation status (DHN-MA, TBARs, COX-2, HO-1, and GCLM), but also to DNA damage (genotoxicity), inflammatory status (colonic MPO and CRP in serum), and a lesser extent barrier function (permeability: ZO-1, Cl5, and JAM-A) (Fig. 7b). Similarly, additional integration of the metabolomic dataset through DIABLO framework allows to confirm that the closest interconnections refer mainly to haem and lipoperoxidation status and are strongly associated with host-bacterial co-metabolic products related mainly to medium- and short-chain fatty acids, products of protein degradation pathways, and hypoxanthine (Fig. 7c).

## Discussion

This study designed to address the short-term impacts of dietary haem iron on the gut barrier revealed that inflammation, permeability, and genotoxicity are increased in the colonic mucosa and closely associated with a profound shift in the gut microbiome. All these changes are prevented by limiting haem iron bioavailability. The haem concentration used in diets (1.5 mmol/g of diet) was physiologically relevant because it corresponds to the haem content of beef meat and is close to the haem content of a meat-based Western diet [42]. As all diets were balanced for iron concentration, the measured effects directly resulted from the iron form (haem vs. ferric citrate) rather than the quantity of iron administered.

The in vivo and ex vivo results allowed us to conclude that short-term exposure to haem iron increased not only colonic inflammation, as evidenced by increased myeloperoxidase activity and pro-inflammatory cytokines, but also DNA damage and gut permeability. Elinav and others defend that inflammation is causally related to some forms of cancer development, through processes that involve genotoxicity via the genotoxic activity of reactive oxygen produced by tissue macrophages and neutrophils, or through producing pro-inflammatory cytokines such as IL-1β [43]. In agreement with these findings, red meat intake is associated with colorectal carcinogenesis and increased inflammatory biomarkers, such as plasma C-reactive protein [1, 44], and increased faecal water genotoxicity [45]. Increased gut permeability is worth noting as it has not yet been explored, in the context of red meat consumption or colitis-associated cancer.

The present study highlights the direct role of aldehydes in short-term haem-induced alterations of gut barrier homeostasis. Ex vivo trapping of aldehydes and the limitation of aldehyde production in vivo by the addition of calcium carbonate to the diet were sufficient to prevent deleterious effects associated with haem iron/peroxidation coupling, including inflammation, genotoxicity, and permeability. These results related to lipoperoxidation agree with previous in vitro studies showing that alpha and beta unsaturated aldehydes (alkenals) can enhance cellular inflammatory processes [11], and in vivo long-term studies addressing haem-induced lipoperoxidation in the promotion of colon carcinogenesis [8].

Evaluation of the luminal side through ^1^H-NMR-based metabolomics reinforced by gas chromatography and microbiota composition analysis demonstrated that the replacement of ferric citrate by haem iron in the diet induced a profound shift in the composition and functionality of the microbiota that strongly correlated with lipoperoxidation status.

In an absence of changes in the carbohydrate content between the experimental diets, the sharp increase in SCFA production and 5-aminovalerate most likely results from protein fermentation and amino acid catabolism due to bacterial modification induced by haem bioavailability. Several amino acids are known precursors of SCFA production by bacterial metabolic activity [46]. A similarly marked increase in the colonic content of SCFAs and isovalerate in rats fed with a high-protein diet has been reported [47]. Furthermore, Van Hecke et al. have shown that in vitro digestion of meat results in approximately twofold higher concentrations of acetate and propionate and threefold higher concentrations of butyrate compared to the blank digests [48]. Interestingly, we also observed a significant increase in faecal trimethylamine contents upon HEM treatment and a tendency to normalise the trimethylamine levels upon calcium addition. Trimethylamine has been identified as an intestinal bacterial metabolite of l-carnitine, a pathway that has recently been shown to be more abundant upon red meat digestion as compared to white meat [49, 50]. Trimethylamine can be subsequently converted into trimethylamine-*N*-oxide in the liver, a compound that has been causatively linked to atherosclerosis [51]. A recent prospective cohort study also revealed that elevated plasmatic trimethylamine-*N*-oxide levels are associated with higher incidence of colorectal cancer in post-menopausal women [52].

Regarding the associated microbiota composition in the literature, meat intake in humans is characterised by an increased abundance of *Bacteroidetes*, including *Bacteroides* and *Alistipes* genera and *Bilophila wadsworthia*, whereas the number of taxa belonging to *Firmicutes* and *Clostridia* is decreased [53, 54]. In mice, dietary haem decreases α-diversity and increases the *Bacteroidetes* to *Firmicutes* ratio and the abundances of *Proteobacteria* [55, 56]. Similar changes at the phylum, family, or genus level induced by the haem iron diet were observed in our study (detailed in Additional file 2: Figure S7). As shown in humans [57], propionate levels were associated with *Bacteroidetes* abundance, probably through the succinate pathway. Propionate may also result from the propanediol pathway [58], which is notably used by *Proteobacteria*, including *E. coli*, i.e. communities that were strongly increased in our study in response to haem iron. Interestingly, the genome of adherent-invasive *E. coli* strains (AIEC) frequently found in IBD is enriched in pathways dedicated to propanediol use and iron uptake [59].

The haem iron diet also led to an increase in additional bacterial taxa already described as opportunist pathogens or associated with inflammation and adenoma or colorectal cancer, as detailed in Additional file 2: Figure S5 and S8. Conversely, this dysbiotic signature is characterised by the loss of protective gut commensal strains (belonging to *Lachnospiraceae*, *Ruminococcae*, and *Lactobacillaceae*, Additional file 2: Figure S8), as previously noted in studies including patients with IBD or CRC and in CRC rodent models [60]. Similarities in bacterial features associated with colorectal diseases, and particularly the bloom of pathobionts [61], highlight the potential role of haem iron in CRC initiation by promoting bacteria capable of competing for siderophore-mediated haem iron acquisition and by driving a dysbiotic environment known to trigger colitic and procarcinogenic host responses [62]. However, one can hypothesise that the common feature of the dysbiotic signature observed between IBD/CRC patients and the haem iron-fed rats may result from the presence of blood in the stools. Nonetheless, by colonising germ-free mice with faeces from CRC patients or healthy volunteers, Baxter and colleagues showed that the imbalance of CRC microbiota triggered the higher tumour occurrence in mice subjected to AOM/DSS treatment [63]. However, the changes in microbiota over the course of the AOM/DSS model were equivalent in both groups, and the final tumour occurrence may be attributed to the altered CRC microbiota rather than to the presence of blood in stools.

Interestingly, genera known as mucin degraders were enriched in mice colonised with CRC-associated faecal microbiota. Accordingly, higher abundance of the *O*-glycan-degrading species *B. massiliensis* [64], members of the genus *Alistipes* and *Parabacteroides*, as well as the sulphate-reducing/H_2_S-producing genus *Desulfovibrio* [65] was associated with haem exposition (detailed in Additional file 2: Figure S9). In addition to its mucin denaturing capabilities, the amino acid-derived hydrogen sulphide (H_2_S) produced by *Desulfovibrio* spp. inhibits colonocyte respiration when present in excess [66], thus representing a “metabolic troublemaker” against epithelium [67]. These results are in accordance with the model of haem-induced mucolysis proposed by Ijssennagger et al. [68]. Haem-induced depletion of *Mucispirillum schaedleri*, a species with a mucus-associated niche in the distal colon and whose abundance would be related to the thickness/robustness of the mucus network, has also been reported [65].

Taken together, these results reinforce the hypothesis of a defect in the mucus barrier in response to haem exposure, leading to facilitated access to the mucosa for both deleterious luminal haem-induced compounds and opportunistic pathogens, able to collectively promote epithelial permeability, inflammation, and genotoxicity. Homeostasis disruption due to haem iron intake at a nutritional dose seems to be explained largely by aldehydes produced by haem-induced lipoperoxidation.

Individual contributions to lipid peroxidation and the ability to trigger DNA damage of bacterial taxa positively correlating with the lipoperoxidation status in our study will have to be explored. In vitro, some commensal and probiotic *E. coli* strains have already been shown to induce lipid peroxidation in liposomes [69] or DNA damage [70].

Increased in vivo fitness of these lipoperoxidation-dependent communities inversely correlated with a loss of protective communities, such as *Lactobacilli* [71] *and Clostidiales* [72] belonging to *Ruminococcaceae*. Poor resistance to haem-induced active ROS due to the lack of catalase activity may be one reason for their reduced relative abundance [73]. Interestingly, several *Lactobacillus* strains reduce intestinal absorption of dietary purines, including hypoxanthine. Accordingly, we found that faecal hypoxanthine levels were negatively correlated with DHN-MA and TBARs and positively correlated with *Lactobacilli* occurrence. Lower levels of hypoxanthine are associated with higher occurrence of colorectal cancer [74].

Finally, our results allow us to propose that short-term exposure to haem iron induces alterations in main actors in the colonic gut barrier through lipid peroxidation. However, the capacity of *Lactobacilli* to prevent lipid peroxidation in vitro [75] and in vivo [76] through antioxidative processes has been described, suggesting that restoring *Lactobacilli* levels through probiotic treatment would be an efficient way to limit haem iron-induced gut barrier defects and probably prevent long-term carcinogenesis.

## Conclusion

We have demonstrated that consumption of a haem-enriched diet results in the alteration of gut microbiota composition and function that was associated with gut barrier defects. Bacterial dysbiosis in response to haem displays common feature with the signatures encountered in colorectal carcinogenesis. Our findings indicate a causal link between haem-induced aldehyde levels in faecal water and gut barrier defects and covariations between these aldehydes and breakdown in microbiome homeostasis. Defining the complex activity of dietary haem iron on the gut barrier actors as a whole will pave the way for nutritional interventions that would limit the formation and effects of haem-induced aldehydes, consequently preventing the risk of colon carcinogenesis associated with the consumption of haem-rich meats.

## Additional files

Additional file 1: Experiment raw data and tables. (XLSX 167 kb)

Additional file 2: Additional figures. (PDF 1924 kb)
