# Supplementary material for: Haem iron reshapes colonic luminal environment: impact on mucosal homeostasis and microbiome through aldehyde formation
Source: Microbiome. 2019 May 6;7:72. doi: 10.1186/s40168-019-0685-7 (PMC6503375; doi:10.1186/s40168-019-0685-7)
Supplement: Supplementary file 2 — Additional figures. (PDF 1924 kb) [file 40168_2019_685_MOESM2_ESM.pdf]

Haem iron reshapes colonic luminal environment: impact on mucosal homeostasis and microbiome through aldehyde formation

Martin O.C.B.[1,4*], **Olier M.**[1*,#], Ellero-Simatos S.[1], Naud N.[1], Dupuy J.[1], Huc L.[1], Taché S.[1], Graillot V.[1], Levêque M.[1], Bézirard V.[1], Héliès-Toussaint C.[1], Blas Y Estrada F.[1], Tondereau V.[1], Lippi Y.[1], Naylies C.[1], Peyriga L[3], Canlet C[1], Davila A.M.[2], Blachier F.[2], Ferrier L.[1], Boutet-Robinet E.[1], Guéraud F.[1], Théodorou V.[1], Pierre F.H.F.[1,#]

Additional file 2

Figure S1: *In vivo* effects of haem-enriched diet (Study 2)
Figure S2 ^{1}H-NMR analysis of faecal extracts
Figure S3 Integrative analysis of metabolomic and physiological datasets
Figure S4 LDA scores of LEfSe analysis
Figure S5 Relative abundance of detected faecal opportunistic pathogens in response to haem enriched diet
Figure S6 Integrative analysis of 12 bacterial taxa with physiological and metobolimic datasets
Figure S7 Relative abundance of haem-exposed haem taxa in rats previously described as associated with consumption of an animal-based diet in human, meat in human and haem in mice
Figure S8 Relative abundance of haem-exposed bacterial taxa belonging to previously identified CRC bacterial signatures in human as compared to healthy volunteers or models of CRC in rodent
Figure S9 Behaviour of mucus associated bacterial taxa in response to haem exposure

Figure S1

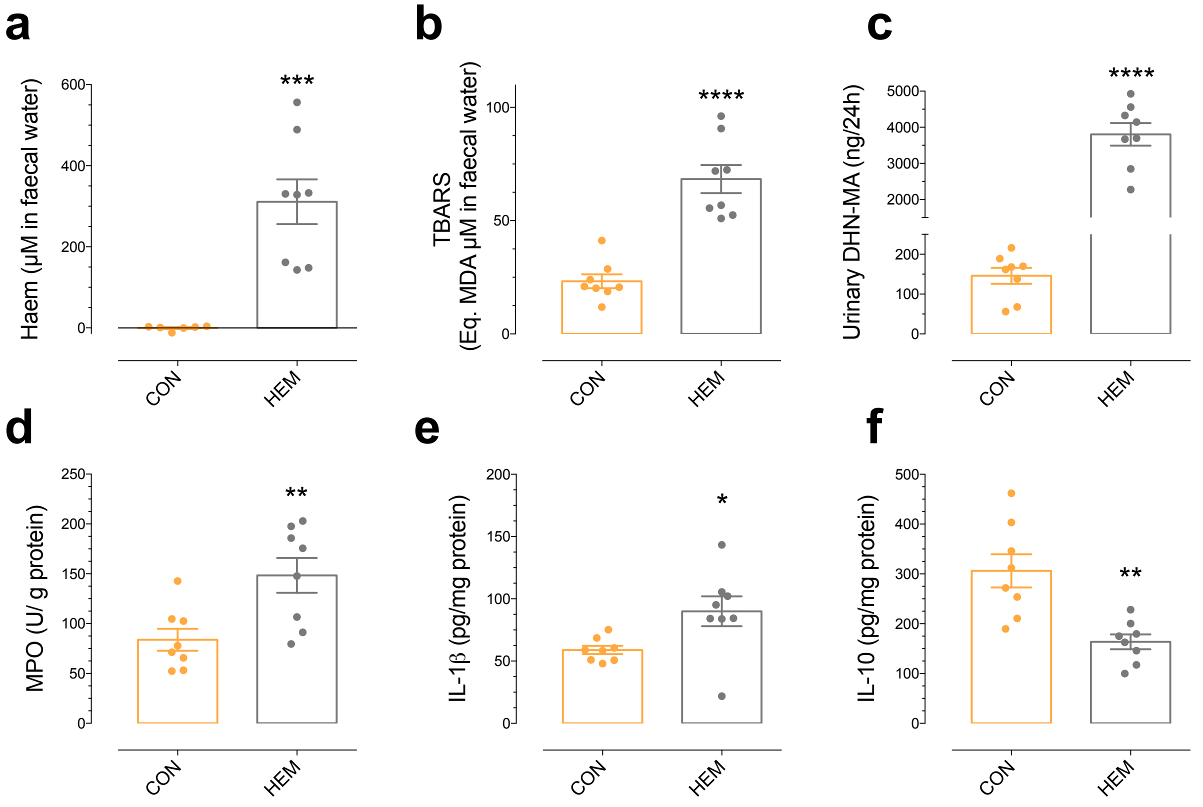

Fig. S1 *In vivo* effects of haem-enriched diet (Study 2). After 14 days of experimental diet, impact on (**a**) faecal haem, (**b**) faecal TBARs, (**c**) urinary DHN-MA, (**d**) colonic myeloperoxidase activity, (**e**) IL-1β, and (**f**) IL-10 in colon mucosa. CON: control diet, HEM: haem-enriched diet. Values are presented as means ± SEM; n= 8. ***P<0.001, **P<0.01, *P<0.05 compared to CON by unpaired t-test.

Figure S2

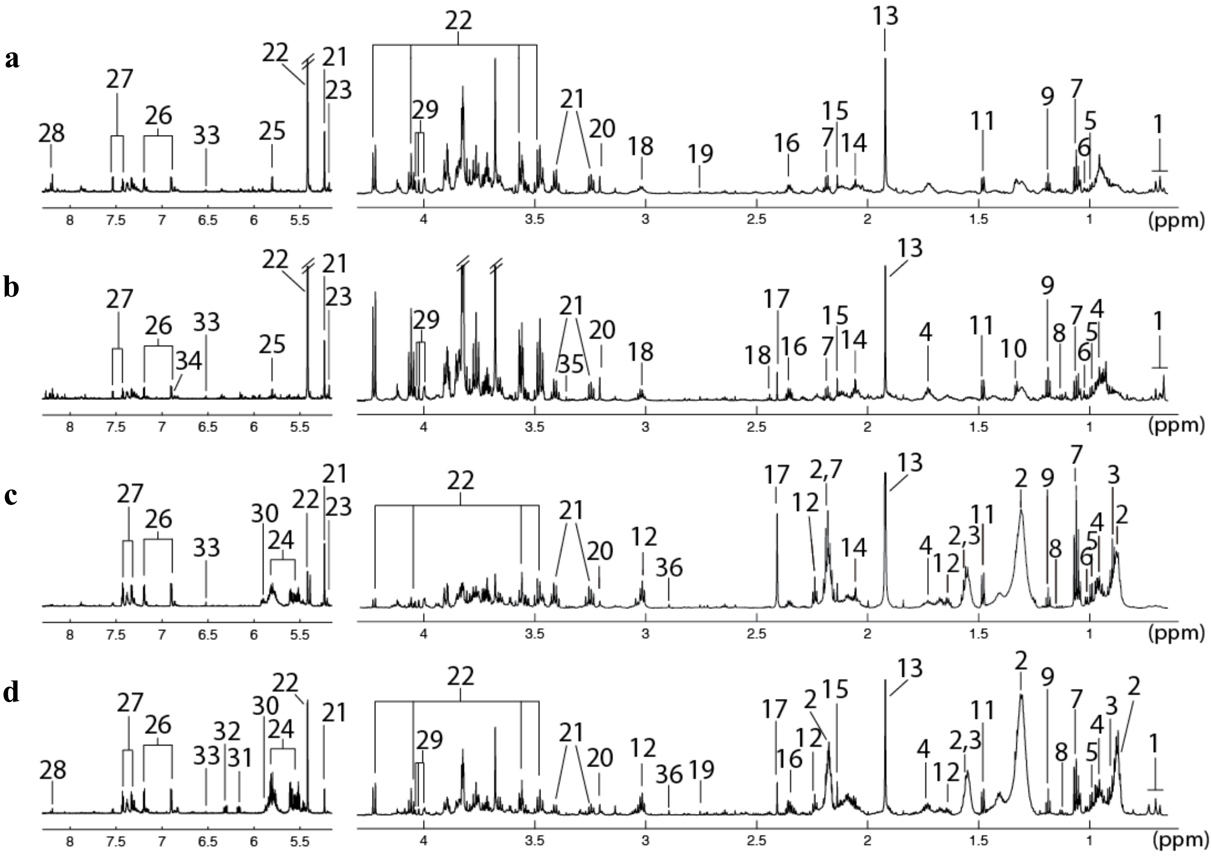

Fig. S2 Typical ^{1}H-NMR spectra of faecal extracts from rats treated with CON (**a**), Ca (**b**), HEM (**c**) or HEM-Ca (**d**).

Key: 1: bile acids (mixed), 2: caprylate, 3: butyrate, 4: leucine, 5: valine, 6: isoleucine, 7: propionate, 8: α-ketoisovalerate, 9: ethanol, 10: lactate, 11: alanine, 12: 5-aminovalerate, 13: acetate, 14: N-acetyl-groups*, 15: methionine, 16: glutamate, 17: succinate, 18: α-ketoglutarate, 19: dimethylamine, 20: choline, 21: β-glucose, 22: sucrose, 23: xylose, 24: unknown compound (U2), 25: uracil, 26: tyrosine, 27: phenylalanine, 28: hypoxanthine, 29: unknown compound (U1), 30: uridine, 31: unknown compound (U3), 32: unknown compound (U4), 33: fumarate, 34: 3-(4-hydroxyphenyl)propionic acid, 35: methanol, 36: trimethylamine.

Figure S3

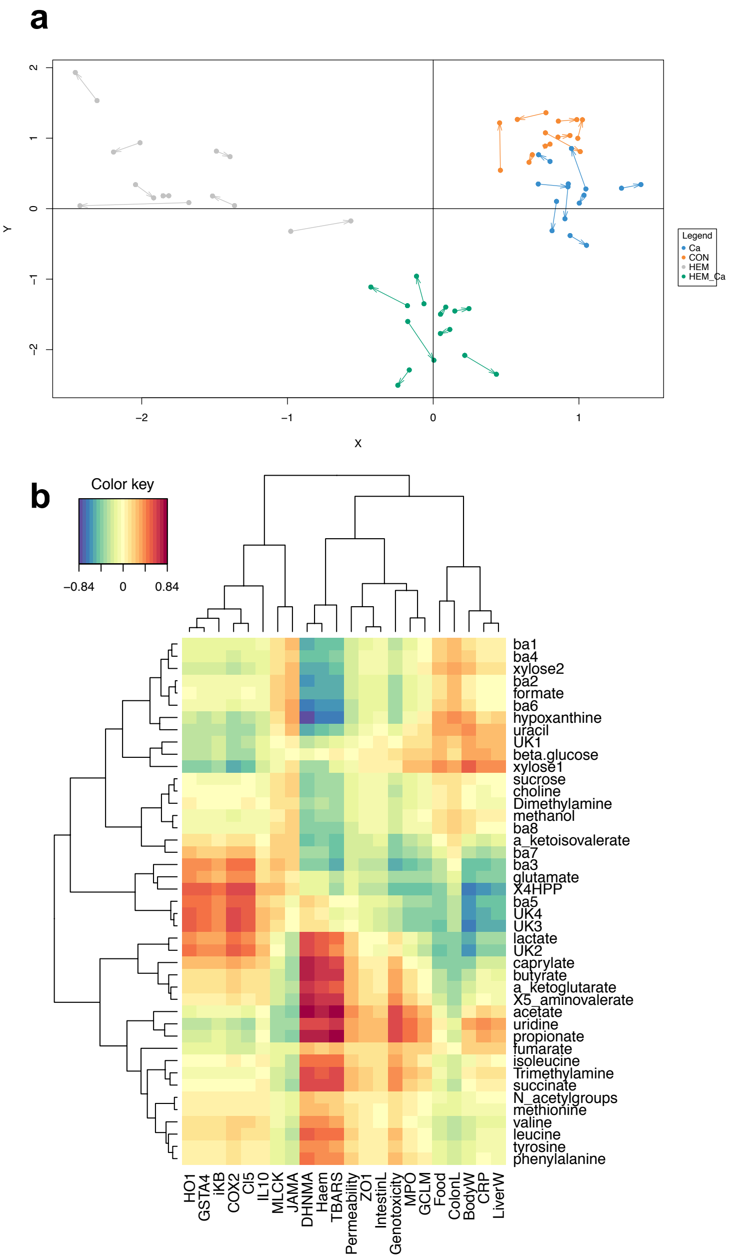

Fig. S3 rCCA aiming at maximising the correlation between metabolomic data and physiological metadata. **a** Plotarrow representing the projection of each sample (individual rat) through an arrow from the metabolome subspace (the start of the arrow) to the metadata subspace (physiological traits indicated by the tip of the arrow) spanned by the two first components retained in the analysis. Length of the arrow indicates the proximity of data structuration between the two datasets. **b** Clustered Image Map (CIM) of the variables from the two datasets. The red and blue colours indicate regions where metabolites and metadata highly positively and negatively correlated, respectively.

HO1: Haem Oxygenase1, GSTA4: glutathione S-transferase alpha 4, iKB: ikappaB, COX2: cyclooxygenase2, Cl5: claudin 5, IL10: IL10 in serum, MLCK: myosin light-chain kinase, JAMA: junctional adhesion molecule, DHNMA: urinary 1,4 dihydroxynonenal, Haem: haem in faecal water, TBARS: faecal thiobarbituric reactive substances, Permeability: Cr51 permeability, ZO1: tight junction protein 1, IntestinL: intestine length, Genotoxicity: DNA damage by comet assay, MPO: myeloperoxidase, GCLM: glutamate-cysteine ligase modifier, Food: food intake, Colon L: colon length, BodyW: body weight, CRP: C-reactive protein, LiverW: liver weight.

Figure S4

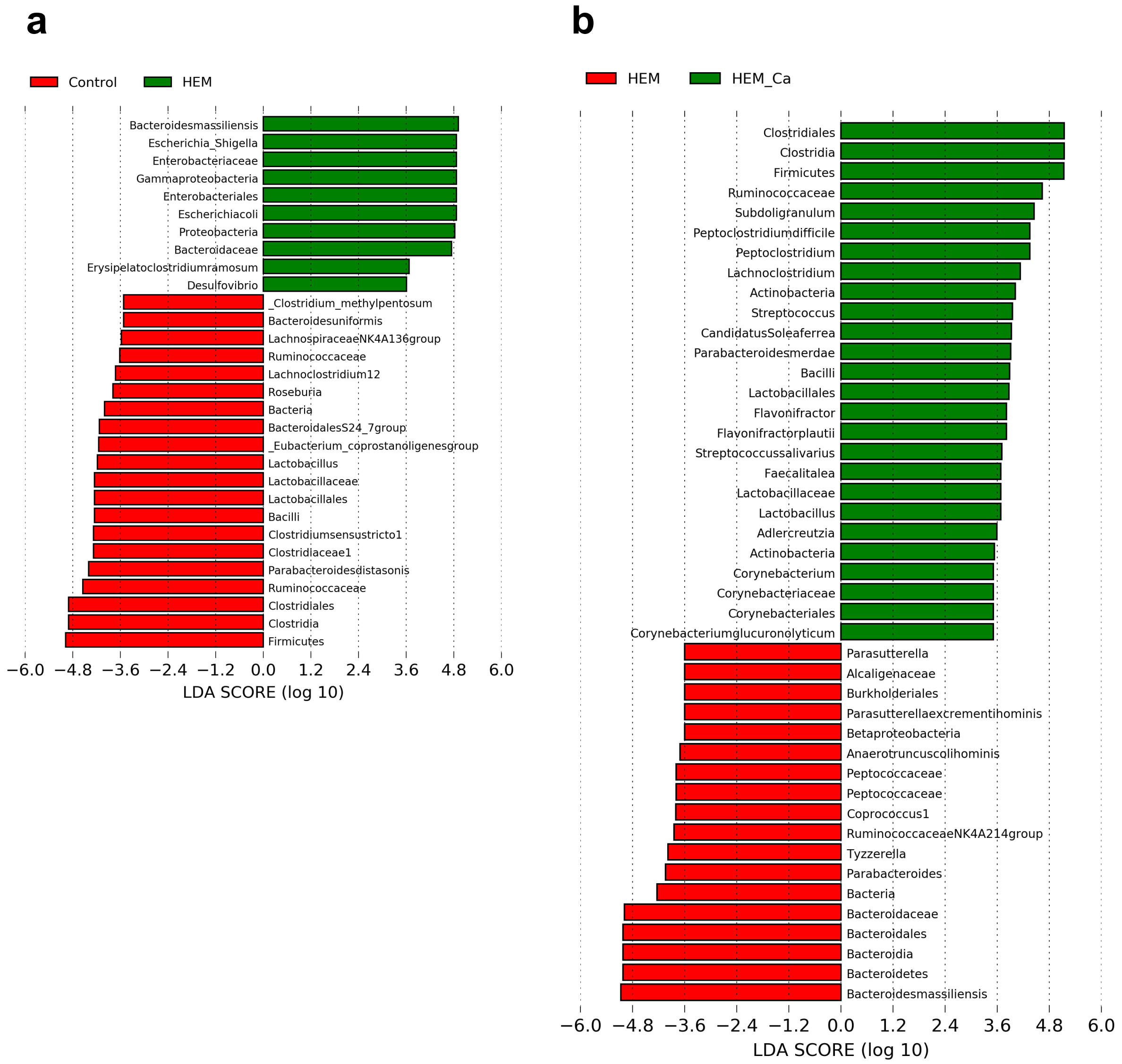

Fig. S4 Histogram of the LDA scores computed for taxa that were found to be differentially abundant at between experimental conditions : **a** HEM vs. CON, **b** HEM_Ca vs. HEM. The magnitude of the LEfSe scores indicates the degree of consistency of the difference, only taxa with LDA scores higher than 3.5 and P<0.01 are displayed.
Differences are represented in the colour of the most abundant class.

Figure S5

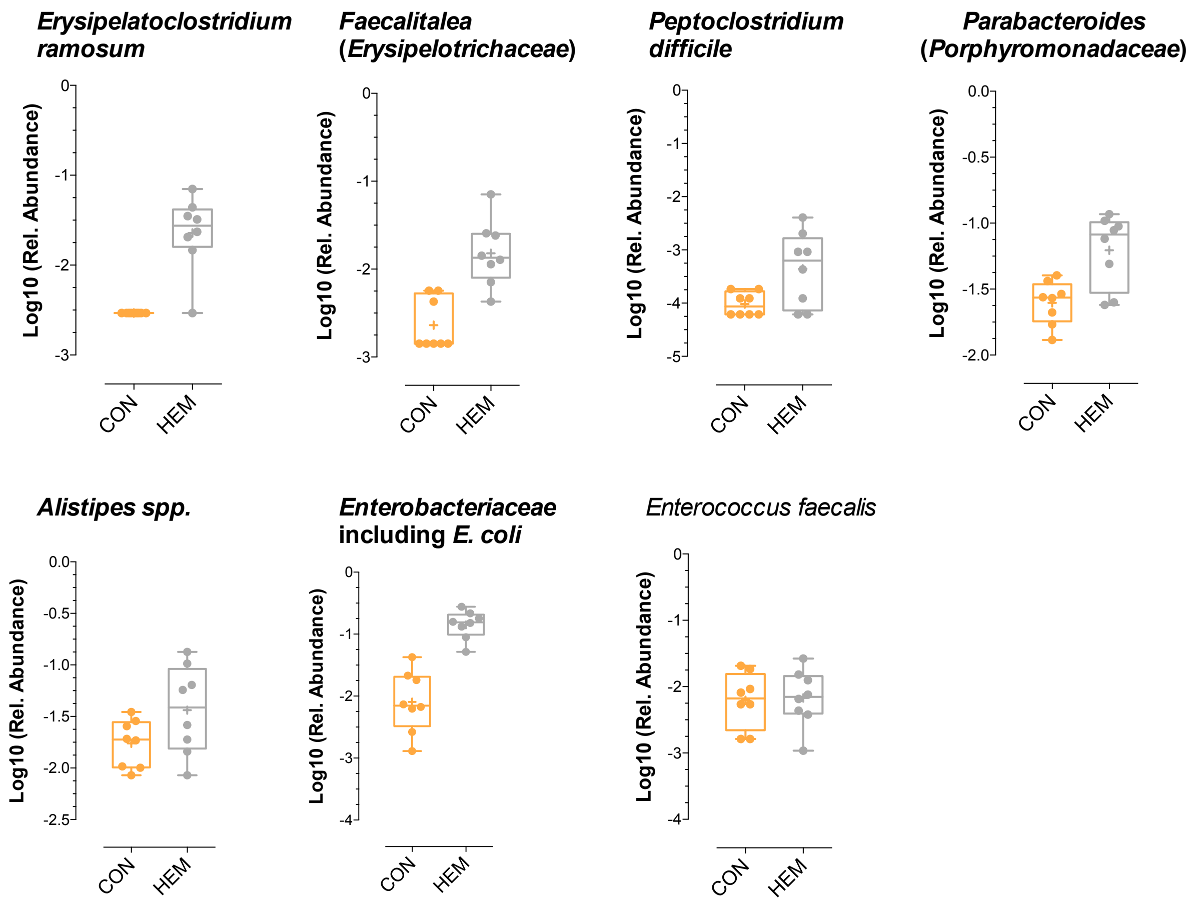

Fig. S5 Relative abundance of detected faecal opportunistic pathogens in response to haem enriched diet. Besides *E. fecalis* abundance, all detected opportunistic pathogens are increased in haem-enriched diet (in bold). CON: control diet and HEM: haem-enriched diet.

Figure S6

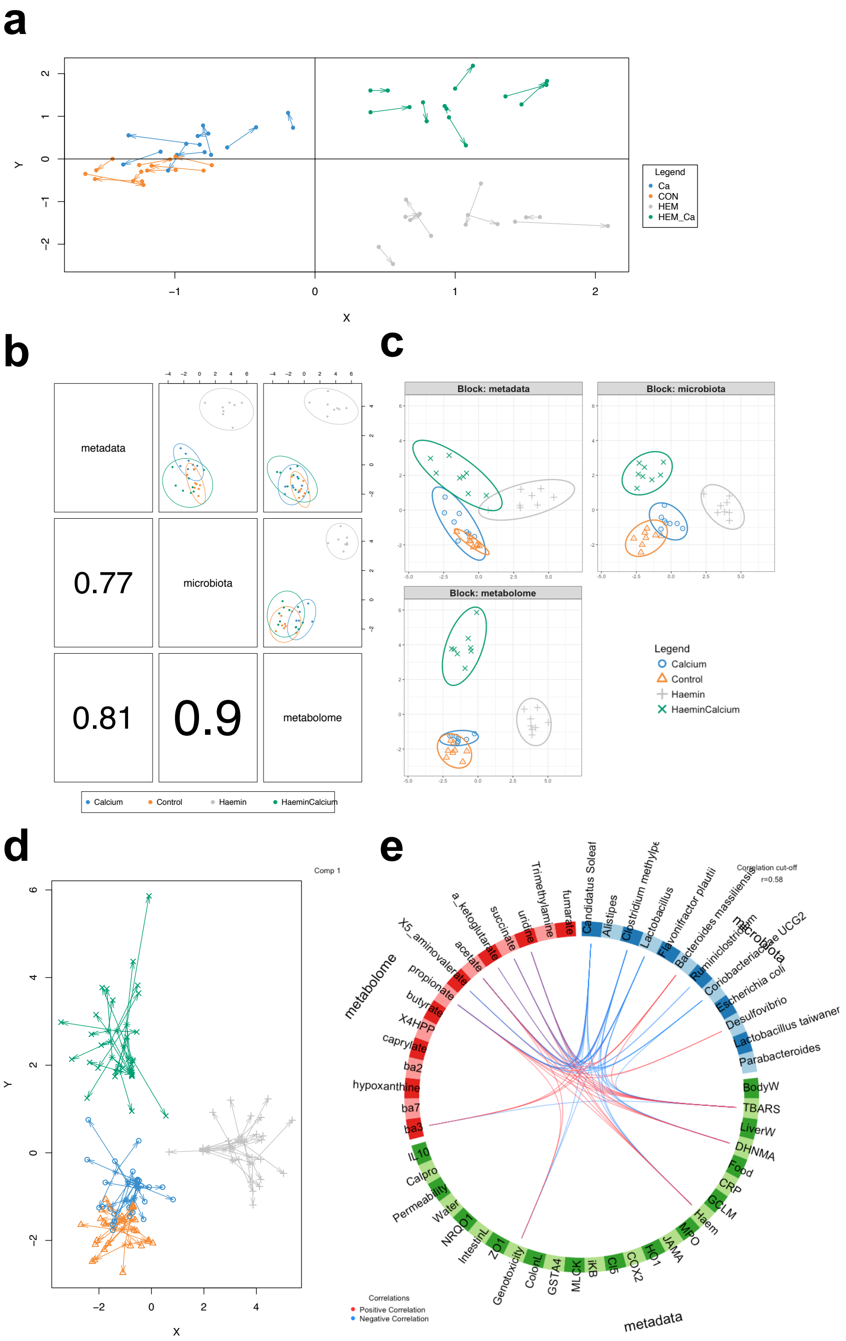

Fig. S6 Multivariate integrative analysis aiming at maximising the correlation between two (a) or three datasets (b-e) . **a** Plotarrow representing the projection of each sample (individual rat) through an arrow from the microbiota subspace corresponding to the 12 selected bacterial taxa (the start of the arrow) to the metadata subspace (physiological traits indicated by the tip of the arrow) spanned by the two first components retained in the analysis. **b** Matrix scatterplot on the 2 first components of each data set depicting the correlations between data sets. **c** Sample plot depicting the clustering of individuals according to diet into the space spanned by the two first components of each block. **d** Plotarrow representing the projection of each sample (individual rat) through the three subspaces (the start of the arrow indicates the centroid between all datasets and the tip of the arrows the location of each sample in each block). **e** Circos plot depicting the higher correlations (cut-off>0.58, component 1) between variables across the three data sets.

Length of the arrow in **a** and **d** indicates the proximity of data structuration between the data sets.

Figure S7

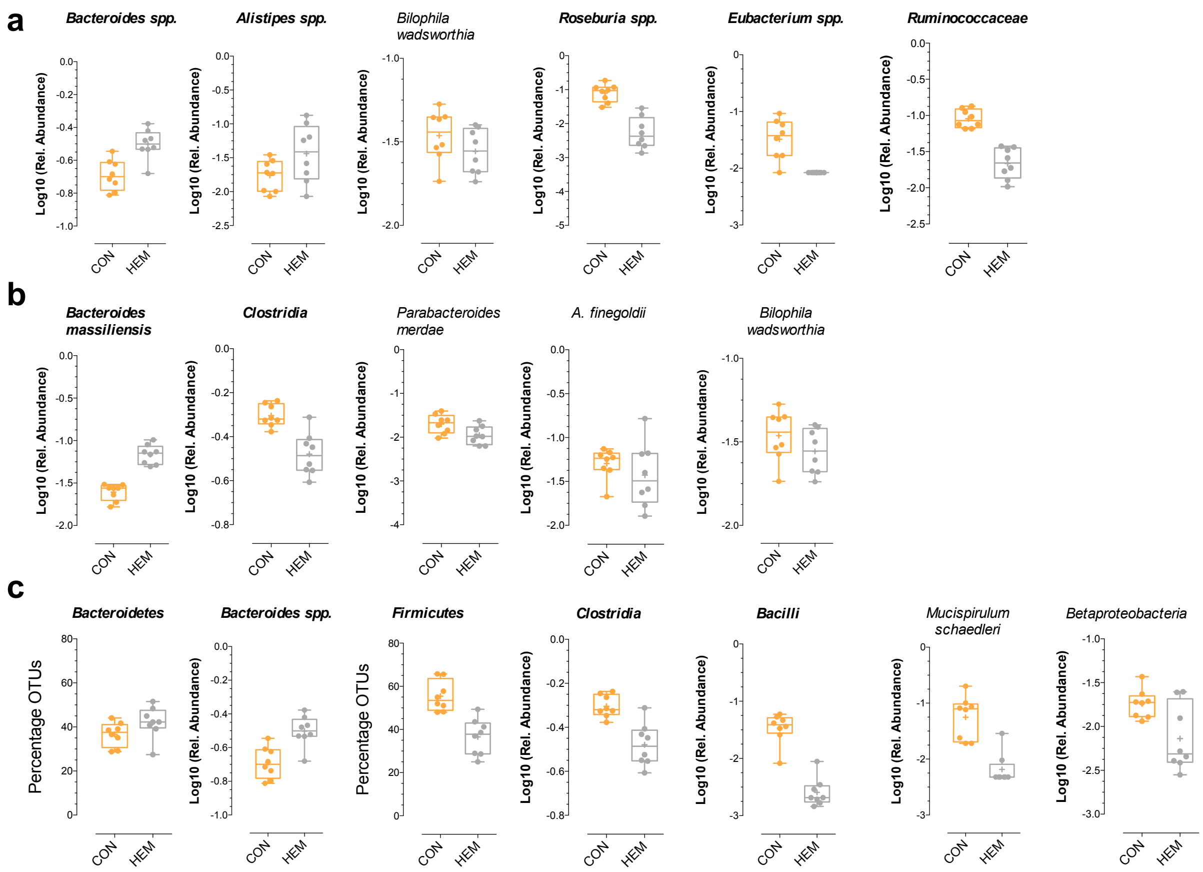

Fig. S7 Relative abundance of haem-exposed haem taxa in rats previously described as associated with consumption of (**a**) an animal-based diet in human [1], (**b**) meat in human [2] and (**c**) haem in mice [3]. Name of the taxa for each graph appeared in bold when in accordance with literature.
CON: control diet and HEM: haem-enriched diet.

Figure S8

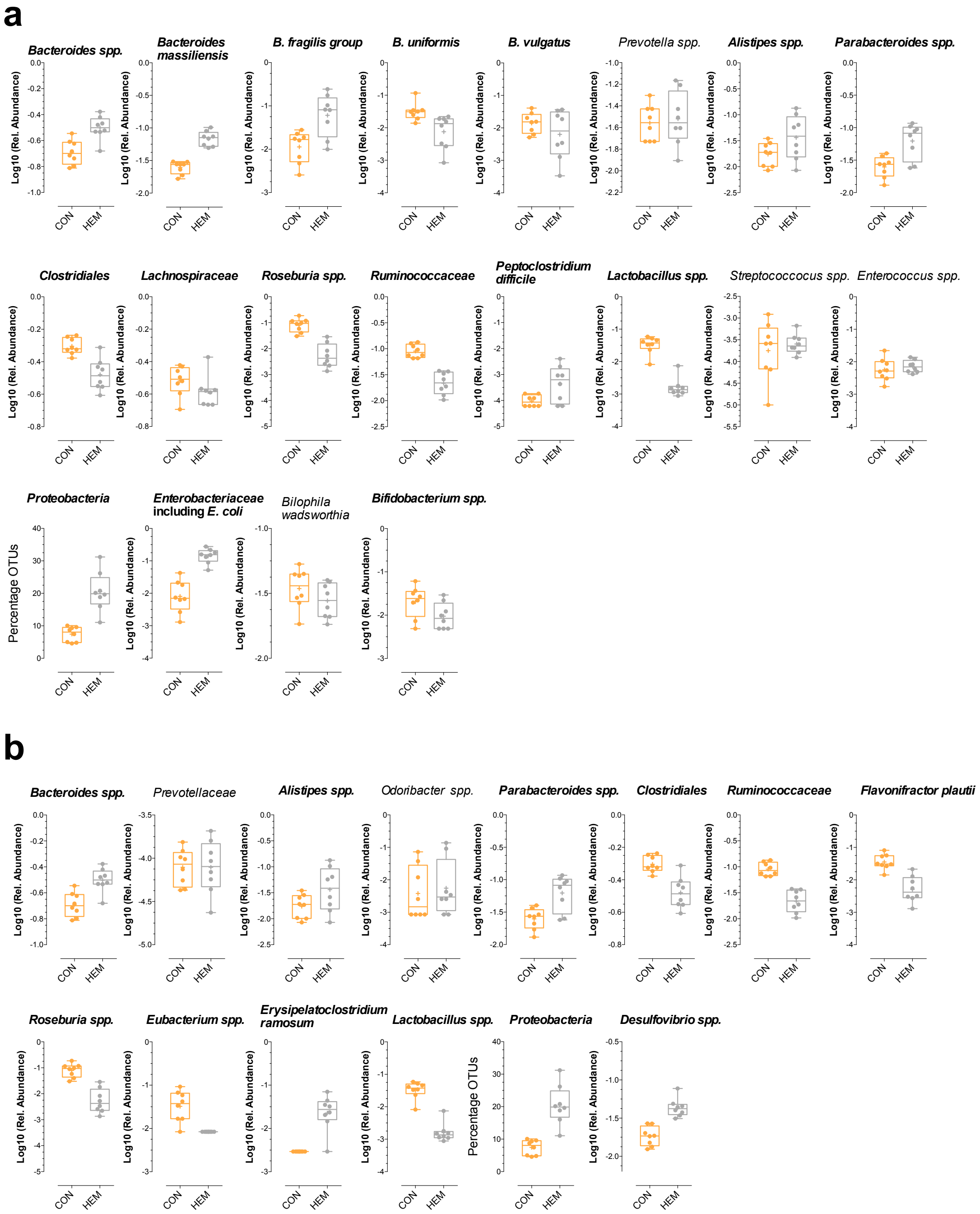

Fig. S8 Relative abundance of haem-exposed bacterial taxa belonging to (**a**) previously identified CRC bacterial signatures in human as compared to healthy volunteers or (**b**) models of CRC in rodent.

Comparison of bacterial relative abundances with these previously described in human were performed only if sufficient number of Genus/Family/Orders/Class/Phylum were detected in microbiome of the studied rats (Taxa belonging to Fusobacterium for example were not detected in this study, as previously seen in mice transplanted with human haem microbiota in the study of Baxter et al. [4]. Similarly, *Akkermansia muciniphila* as well as *Campylobacter spp.* or *Streptococcus bovis* were not detected in our study.

Included studies for comparisons were performed on faeces [2,4–9] and comparisons were primarily based on the review from Borges-Canha et al. [10] that considered only consistent changes among human or animal studies.

Name of the taxa for each graph appeared in bold when in accordance with literature.

CON: control diet and HEM: haem-enriched diet.

Figure S9

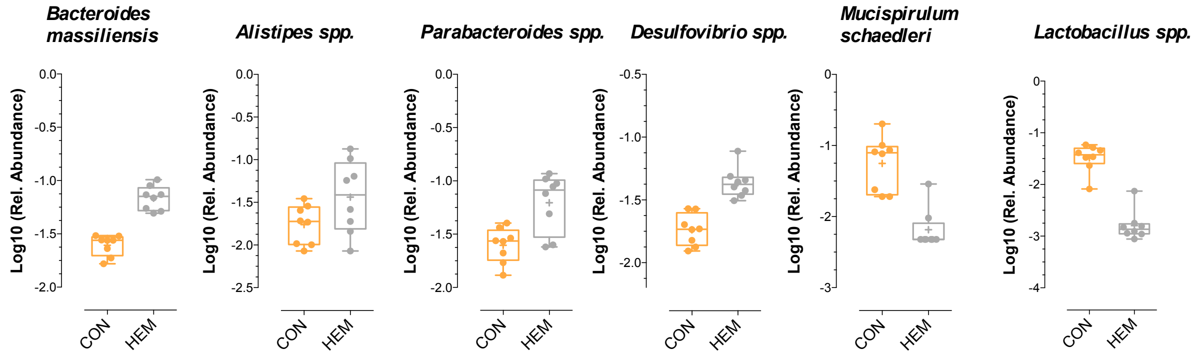

Fig. S9 Behaviour of mucus associated bacterial taxa in response to haem exposure. CON: control diet and HEM: haem-enriched diet.

Additional References

1. David LA, Maurice CF, Carmody RN, Gootenberg DB, Button JE, Wolfe BE, et al. Diet rapidly and reproducibly alters the human gut microbiome. Nature. Nature Publishing Group; 2014;505:559–63.

2. Feng Q, Liang S, Jia H, Stadlmayr A, Tang L, Lan Z, et al. Gut microbiome development along the colorectal adenoma-carcinoma sequence. Nat. Commun. 2015;6:6528.

3. IJssennagger N, Derrien M, van Doorn GM, Rijnierse A, van den Bogert B, Müller M, et al. Dietary heme alters microbiota and mucosa of mouse colon without functional changes in host-microbe cross-talk. PLoS One. 2012;7:e49868.

4. Baxter NT, Zackular JP, Chen GY, Schloss PD. Structure of the gut microbiome following colonization with human feces determines colonic tumor burden. Microbiome. 2014;2:20.

5. Ahn J, Sinha R, Pei Z, Dominianni C, Wu J, Shi J, et al. Human gut microbiome and risk for colorectal cancer. J. Natl. Cancer Inst. 2013;105:1907–11.

6. Wang T, Cai G, Qiu Y, Fei N, Zhang M, Pang X, et al. Structural segregation of gut microbiota between colorectal cancer patients and healthy volunteers. ISME J. 2012;6:320–9.

7. Wu N, Yang X, Zhang R, Li J, Xiao X, Hu Y, et al. Dysbiosis signature of fecal microbiota in colorectal cancer patients. Microb Ecol. 2013/06/05. 2013;66:462–70.

8. Zackular JP, Rogers MAM, Ruffin MT, Schloss PD. The human gut microbiome as a screening tool for colorectal cancer. Cancer Prev. Res. 2014;7:1112–21.

9. Zhu Q, Jin Z, Wu W, Gao R, Guo B, Gao Z, et al. Analysis of the intestinal lumen microbiota in an animal model of colorectal cancer. PLoS One. 2014;9:e90849.

10. Borges-Canha M, Portela-Cidade JP, Dinis-Ribeiro M, Leite-Moreira AF, Pimentel-Nunes P. Role of colonic microbiota in colorectal carcinogenesis: A systematic review. Rev. Esp. Enfermedades Dig. 2015;107:659–71.
